# Polyolefin-Supported Hydrogels for Selective Cleaning Treatments of Paintings

**DOI:** 10.3390/gels6010001

**Published:** 2019-12-18

**Authors:** Silvia Freese, Samar Diraoui, Anca Mateescu, Petra Frank, Charis Theodorakopoulos, Ulrich Jonas

**Affiliations:** 1 Department Chemistry - Biology, University of Siegen, Adolf-Reichwein-Strasse 2, D-57076 Siegen, Germany; silvia.freese@uni-siegen.de (S.F.); samar.diraoui@gmx.de (S.D.); frank@chemie.uni-siegen.de (P.F.); 2 Continental Automotive Romania, Research and Development, Display Technology Department, Strada Siemens 1, 300704 Timisoara, Romania; ancamateescu2001@yahoo.com; 3 Department of Arts, Science in Conservation of Fine Art, Northumbria University, Newcastle upon Tyne NE1 8ST, UK

**Keywords:** thin photocrosslinked hydrogels, flexible polyacrylamide-coated PE sheets, varnish surface solubilization, soil removal, art conservation technology

## Abstract

Surface decontamination is of general concern in many technical fields including optics, electronics, medical environments, as well as art conservation. In this respect, we developed thin copolymer networks covalently bonded to flexible polyethylene (PE) sheets for hydrogel-based cleaning of varnished paintings. The syntheses of acrylates and methacrylates of the surfactants Triton X-100, Brij 35, and Ecosurf EH-3 or EH-9 and their incorporation into copolymers with acrylamide (PAM) and *N*-(4-benzoylphenyl)acrylamide are reported. Photocrosslinked polymer networks were prepared from these copolymers on corona-treated PE sheets, which can be swollen with aqueous solution to form hydrogel layers. The cleaning efficacy of these PE-PAM hydrogel systems, when swollen with appropriate cleaning solutions, was evaluated on painting surfaces in dependence of the PAM copolymer composition and degree of crosslinking. Specifically, soil and varnish removal and varnish surface solubilization were assessed on mock-ups as well as on paintings, indicating that even surfactant-free cleaning solutions were effective.

## 1. Introduction 

The efficient decontamination of surfaces aiming at the removal of small molecular species and particulate contaminants is of critical concern in diverse industries and routine procedures. Prominent examples include cleaning of optical components [1,2], microelectronics [3], medical environments [4,5], and cultural heritage objects [6,7,8,9]. Since the 1980s, diverse pastes and gel systems [10,11,12,13,14,15,16,17] composed of polymers and customized solutions have been employed for art conservation [18]. These liquids are typically comprised of organic solvents [19], aqueous solutions, and microemulsions blended with various reagents [9] and nanomaterials, typically stabilized by surfactants [20]. Advances in macromolecular chemistry and nanotechnology, specifically for applications such as targeted drug delivery [21,22,23], biotechnology [24,25], immunoassay [26], tissue engineering [27], and processing of bioactive substances [28], have impacted the development of these materials [20]. A distinct interest to refine cleaning methods for paintings emerged [29], aiming at the removal of discolored varnishes [30], soils, paint by-products [31,32], and additives [33,34]. Degradation introduces further complexity into the already intricate architecture of the painting, manifested in mechanical, compositional, and associated polarity heterogeneities [35,36,37,38]. A prominent difficulty in existing cleaning procedures is the restriction of the applied liquids to the outermost surface of the artwork, as liquid diffusion into the bulk layers can lead to deterioration of the paint substrate [39,40,41]. This fact has been recently demonstrated by unilateral nuclear magnetic resonance spectroscopy of acrylic emulsions paints [42]. In addition, free surfactants from the cleaning solutions will partially reside on the painting surface [43] and will have to be removed by application of a rinsing liquid [44].

In order to address these problems, we have developed a thin hydrogel coating on a flexible polyolefin backing to deliver a controlled amount of liquid during the cleaning treatment. Surfactant monomers were copolymerized with hydrophilic monomers to ensure covalent immobilization of the amphiphilic species in the hydrogel network, preventing their leaching during the cleaning procedure. These incorporated surfactant moieties are expected to perform like the free surfactant micelles based on the documented hydrophobic aggregation of apolar groups in hydrophilic polymers [45,46,47,48].

In the following discussion, we present the synthesis of the polyethoxylate surfactant monomers and their copolymerization with acrylamide, including photocrosslinkable monomers, to yield the hydrogel precursors. The preparation procedure for the hydrogel sheets on polyethylene backings by photocrosslinking of the precursor polymers will be described. Examples of preliminary implementation tests on mock-ups and genuine paintings are demonstrated as proof-of-concept.

## 2. Results and Discussion

### 2.1. Concept of Polyolefin-Supported Hydrogels

Our approach to control the liquid exposure of painting surfaces during cleaning treatments involves confinement of such liquids to thin hydrogel films. In order to ensure the required mechanical robustness and provide convenience of handling, such hydrogel layers are covalently bonded [49] to flexible and transparent polyolefin backings. Here, we specifically utilize photocrosslinked polyacrylamide (PAM)-based hydrogels attached to polyethylene (PE) sheets as support.

**Figure 1:**
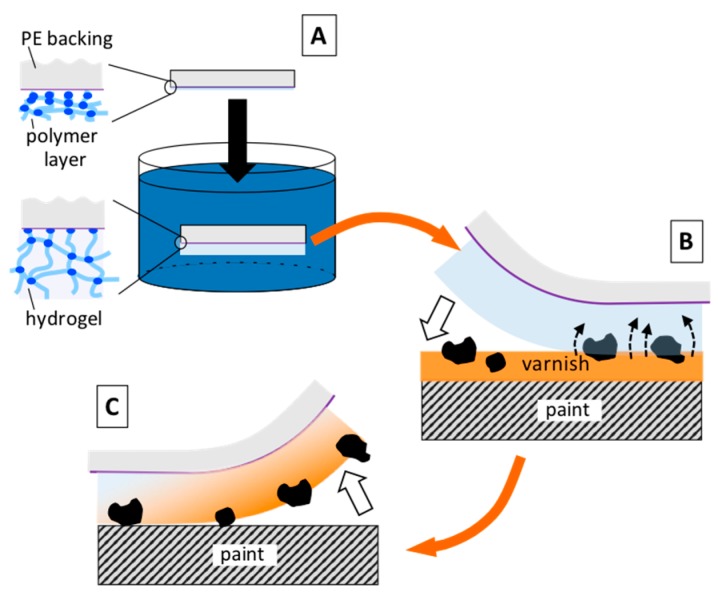
Schematic workflow of the treatment procedure for paintings with polyethylene-supported polyacrylamide (PE-PAM) sheets. (**A**) Swelling of the crosslinked PAM network on the PE backing by immersion into a cleaning solution to form the active hydrogel layer. (**B**) Application of the swollen PE-PAM sheet onto the artwork surface, which solubilizes surface coatings and immobilizes soiling particles on the hydrogel. (**C**) Peeling of the PE-PAM sheet after sufficient incubation time with concomitant removal of detached and dissolved entities.

The cleaning procedure is schematically outlined in Figure 1. In the first step, these PE-supported polyacrylamide (PE-PAM) networks are immersed in aqueous solutions to form the active hydrogel film prior to contact with the painting surface. The solution swells the dry PAM layer, which serves as a liquid reservoir. During the incubation step, surface contaminants or varnish layers will be mobilized and solvated by the liquid from the hydrogel. Dissolved species will diffuse into the swollen polymer network, while mobilized soil particles will stick to the hydrogel surface. With the removal of the PE-PAM sheet in the final process step, these entities will be withdrawn from the painting surface. Released soils pulled on the surface upon application will remain unbounded and can be readily removed by gently rolling a dry cotton swab as soon as the minute liquid introduced by the gels evaporates. 

The benefit of the treatment procedure developed with the PE-PAM sheet lies in the freedom for the art conservator to customize the composition of the cleaning solution, while taking full advantage of the following assets.

The liquid volume per surface area during the treatment is controlled by the thickness and swelling degree of the hydrogel layer, which are determined by the polymer casting process and the UV dose, respectively. With the layer thicknesses in the micron scale, it becomes possible to operate at the minimum volume that activates the cleaning procedure, avoiding excessive liquid exposure of the painting.

Furthermore, as the crosslinking degree is controlled by the UV exposure conditions during the photocrosslinking step [50,51,52], it directly affects the mechanical modulus [53] and softness of the hydrogel (besides its swelling degree).

The chemical composition of hydrogel network can be precisely tailored by the polymer synthesis and allows integration of specific functionalities. In the present example, surfactant side chains are introduced to enhance the affinity to hydrophobic contaminants and varnish components. As the surfactant moieties are covalently attached to the polymer network, surfactant residues on the painting are avoided. Therefore, no subsequent rinsing step is required, in contrast to conventional cleaning treatments employing dissolved surfactant species.

The flexible, but robust PE sheet supporting the hydrogel layer enables convenient handling independent of the mechanical properties of the hydrogel. Its transparency allows to accurately position the PE-PAM sheet on the painting and to directly monitor the treatment progress. Additionally, it can be cut to the desired size and shape of the targeted surface region.

### 2.2. Monomer and Polymer Synthesis

**Scheme 1:**
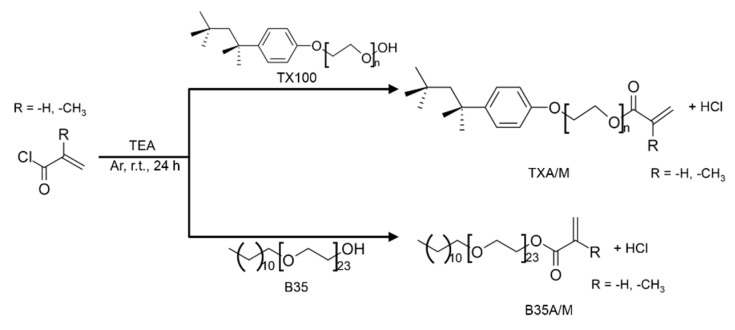
Synthetic pathways for the 4-(1,1,3,3-tetramethylbutyl)phenyl-polyethylene glycol acrylate (TXA), Triton X-100 (TXM), poly(oxyethylene) lauryl ether (Brij 35) acrylate (B35A), and Brij 35 methacrylate (B35M).

In order to covalently immobilize the surfactant moieties in the polymer networks with precise control over the composition, polymerizable acrylate and methacrylate units were introduced into the commercially available parent compounds. As such, the acrylate derivative 4-(1,1,3,3-tetramethylbutyl)phenyl-polyethylene glycol acrylate (TXA) and Triton X-100 methacrylate (TXM) of Triton X-100 (TX100) were obtained by reaction of the hydroxyl group of TX100 with acryloyl chloride and methacryloyl chloride, respectively, employing TEA as the base in accordance with the literature [36]. Analogously, the corresponding monomer derivatives were obtained from the surfactant Brij 35, as depicted in Scheme 1. Details on the Ecosurf EH-n (*n* = 3 or 9) acrylate syntheses are provided in the Supporting Information, Figures S1 and S2.

**Scheme 2:**
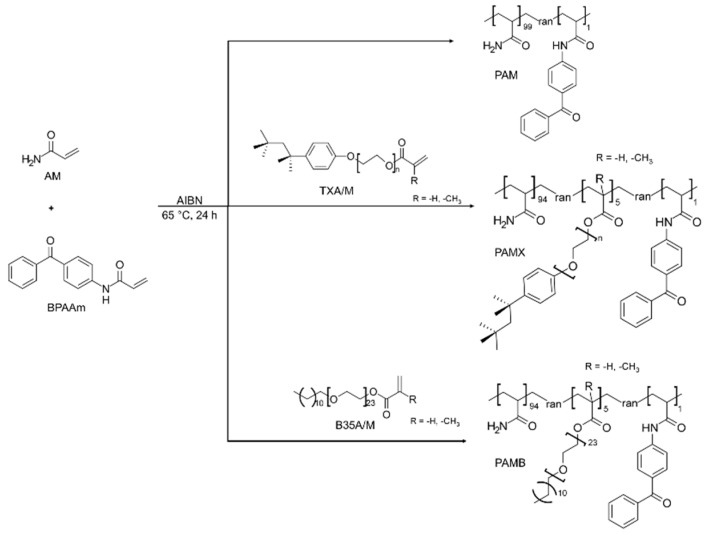
Synthesis scheme for the preparation of PAM copolymers with embedded surfactant moieties.

**Figure 2:**
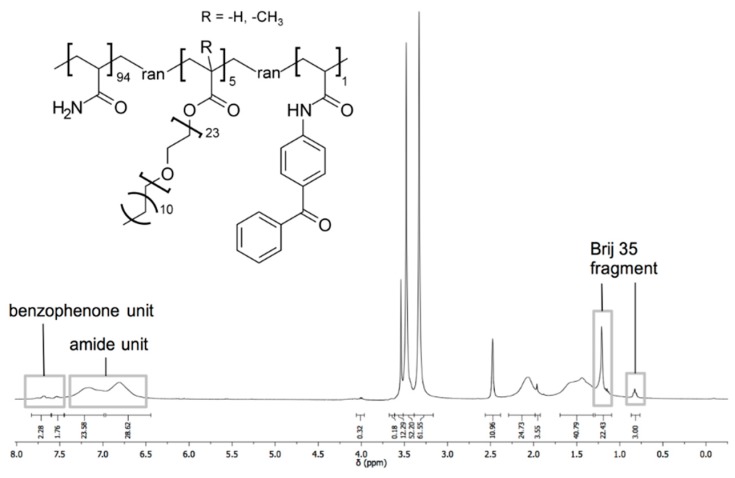
^1^H-NMR spectrum of the PAMB terpolymer with a composition of AM_94_/B35A_5_/BPAAm_1_ in DMSO-D_6_.

These surfactant monomers were employed for free radical copolymerization with comonomer mixtures in dioxane, as shown in Scheme 2. The respective comonomers encompassed acrylamide (AM) as a major constituent endowing hydrophilic character to the polymer for swelling of the hydrogel network, and benzophenone acrylamide (BPAAm) as the photocrosslinking unit. The surfactant comonomers provide amphiphilic characteristics and enhance the affinity of the hydrogel matrix to hydrophobic entities. The copolymer composition was analyzed by ^1^H-NMR spectroscopy and indicated that the built-in ratios of the respective monomer types were in accordance with their monomer feed ratios. A representative ^1^H-NMR spectrum is provided for the copolymer PAMB with composition AM_94_/B35A_5_/BPAAm_1_ in Figure 2. Further ^1^H-NMR spectra of the surfactant monomers and copolymers are provided in the Supporting Information, Figures S3–S14. Here, the aromatic bands for the photocrosslinking unit around δ = 7.5–8 ppm are well separated from the amide protons δ = 6.5–7.5 ppm to allow composition determination by peak integration. Similarly, the characteristic bands for the lauryl fragment of the Brij 35 unit are clearly identified at around δ = 0.8 ppm and 1.2 ppm to allow their quantification in the terpolymer. Residual solvent peaks result from traces of solvents used prior for synthesis or purification of the polymers and cannot be avoided, as fully dried polymers are not soluble anymore in the appropriate NMR solvent. For all polymer systems containing acrylate surfactant derivatives, similar results were found (further NMR spectra and details on Ecosurf-containing polymers are provided in the supporting information).

For the copolymers containing the methacrylate surfactant derivatives, a low solubility in water (Table 1) was found. This resulted in a low tendency of the crosslinked network to swell and hampered the formation of the active hydrogel layer with the aqueous cleaning solutions. Furthermore, the low solubility impeded wetting of the corona-treated PE sheets with the coating solution. Consequently, these methacrylate systems were not further studied in the conservation cleaning tests.

In one attempt, a tetrapolymer composed of four different monomer types was prepared with methacrylic acid as the fourth comonomer to integrate a polar and ionizable repeat unit into the polymer backbone. The carboxylic acid functionality would also allow further chemical modification of the network. As the resulting tetrapolymer showed low solubility in most common solvents (as indicated in Table 1), it was not further utilized in the PE-PAM sheet preparation.

### 2.3. Preparation Procedure for the PE-PAM Sheets 

**Figure 3:**
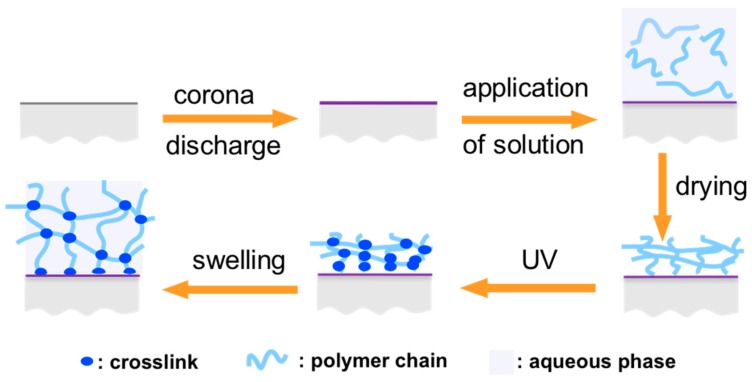
Sequence of process steps for the preparation of PE-PAM sheets.

The workflow for the PE-PAM sheet preparation is presented in Figure 3, starting with a corona discharge treatment of the PE substrate. By this process step, hydrophilic surface moieties are introduced to improve wetting with the polymer solution and adhesion of the PAM layer. Casting of the various PAM types from the water–ethanol solution (1:1 *v*/*v*) was performed by doctor blading, yielding a layer thickness of about 3–6 micrometers (from AFM measurements and optical microscopy, Supporting Information, Figures S16 and S16) after solvent evaporation. Network formation and concomitant bonding of the PAM chains to the PE support was induced by irradiation with UV light at a wavelength of 365 nm for time periods of 10 to 60 min. The specific irradiation time and the exact polymer composition is indicated in Table 2 with the corresponding sample acronyms. Exposure of the photocrosslinked PE-PAM sheets to aqueous solutions swells the polymer network and yields the active hydrogel coating, as described above in Figure 1.

### 2.4. Stability Tests of PE-PAM Sheets

The quality of the adhesive bond between the different PAM layer types and the PE backing was assessed by recording the pulling force required to peel off an adhesive tape from the PE-PAM sheet surface. Good layer bonding was indicated if the adhesive tape came off the surface without detachment of the PAM layer from the PE support. Insufficient bonding led to a delamination of the PAM layer from the PE support, which was directly visible as defects in the PAM layer on the PE support and by PAM fragments attached to the adhesive tape. The photograph showing the customized setup is provided in the Supporting Information, Figure S15. Force data were collected over an extended time period with constantly increasing pulling force and averaged over three to eight experimental repetitions. On the plain PE substrate, an average pull-off force of 0.6 N was measured, while for a corona-treated PE surface, a force of about 1.4 N was required for adhesive tape release. This directly reflects the chemical modification of the PE surface by the corona process. On the PAM_15_-coated surface the pull-off force of about 0.5 N was measured close to the plain PE reference. As no PAM detachment was observed, good bonding between the PAM layer and the PE support was demonstrated.

For the PE-PAMB_15_ sample with incorporated Brij 35 surfactant, the average peel-off force increased to 0.9 N without PAM detachment, again corroborating a sufficient PE-PAM bonding. Apparently, the change of surface polarity effected by the surfactant comonomer leads to stronger attachment of the adhesive tape, which may contribute to an improved cleaning efficacy by the enhanced interaction of the hydrogel matrix with soil particles on the painting surface.

### 2.5. Water Loading Tests 

Gravimentric analysis was employed to qualitatively assess the water capacity of the PE-PAM sheets in dependence of the PAM copolymer composition and crosslinking conditions. For the PE-PAM_15_ and PE-PAMB_15_ systems, which were irradiated for 15 min during photocrosslinking, a water load of about 0.005–0.05 mL/cm^2^ was found for the PAM coatings without surfactant. The PAMB system with incorporated Brij 35 moieties showed an increased water load of 0.01–0.2 mL/cm^2^. Even though the Brij 35 surfactant carries hydrophobic lauryl chains, for which a lower affinity to water would be expected, the hydrophilicity of the poly(ethylene oxide) linker in these surfactants apparently contributes to an enhanced water capacity of the PAMB layer.

Preliminary implementation tests: Prior to any cleaning efficacy tests employing the PE-PAM sheets, swab rolling pre-tests were conducted. For these pre-tests, aqueous solutions were specifically prepared to induce the desired effect on the artwork surface, like soil removal from varnish, soil removal from paint, or varnish removal from paint. The solutions were applied to the artwork surface by swab rolling, followed by clearing with a surfactant-free rinsing solution with otherwise same composition.

We recently demonstrated the cleaning efficacy of surface-attached gels for the immobilized soils from aged painting varnishes [54]. In the following section, we present specific examples to highlight the versatility of this novel technology.

#### 2.5.1. Test 1—Soil Removal from Aged Varnish

**Figure 4:**
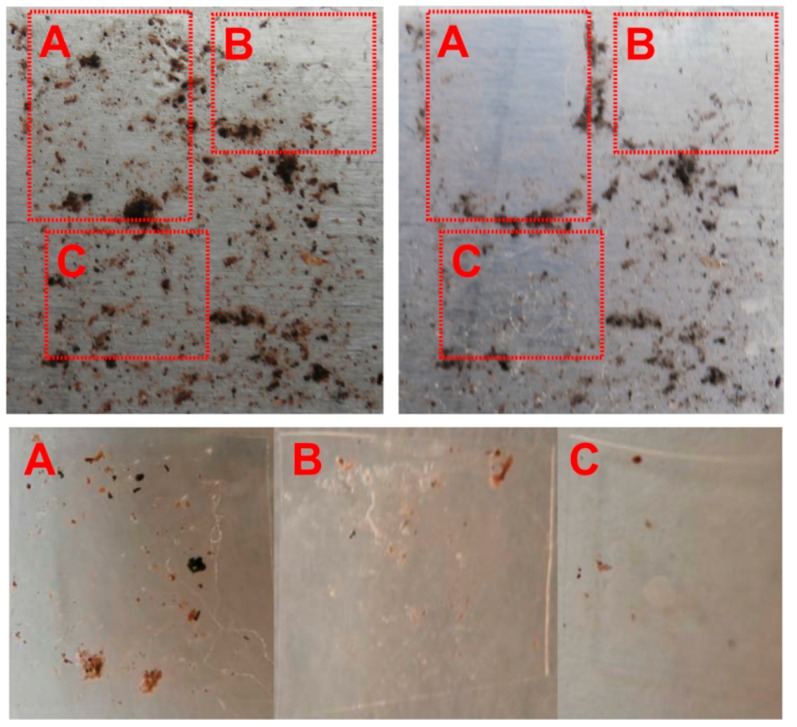
Top panel (image width corresponds to 2.5 cm): Laropal K80 mock-ups, left: before treatment, right: after treatment. Lower panel (image height corresponds to 1 cm): PE-PAM sheets after application. (**A**) PE-PAM_15_, (**B**) PE-PAM_30_, (**C**) PE-PAM_60_.

The capacity to effectively remove soils from varnishes was performed in a first test series with various PE-PAM hydrogel types on soiled and aged Laropal K80-varnish mock-ups. Upon contact, the PE-PAM hydrogel effectively wets the mock-up surface with the cleaning solution. Concomitantly, the soil particles adhere to the hydrogel interface and readily detach from the varnish upon withdrawal of the polymer sheet, leaving a clean and optically unaltered surface. Figure 4 shows that the PE-PAM_15_ hydrogel with the least crosslinking time of 15 min was most efficient in removing the soils from the Laropal K80 varnish surface, while the PE-PAM_30_ hydrogel was less efficient. In contrast, the PE-PAM_60_ system with the longest photocrosslinking time (60 min) failed to sufficiently remove soils from the varnish. The superior cleaning efficacy of the less crosslinked systems corresponds to a stronger swelling of the PAM network with higher liquid loading capacity, resulting in a softer hydrogel matrix. This softness ensures good conformal contact with the rough substrate. The hydrogel systems with incorporated surfactant moieties were more efficient [55]. 

#### 2.5.2. Test 2—Removal of Mastic Varnish from Egg Tempera Paint

**Figure 5:**
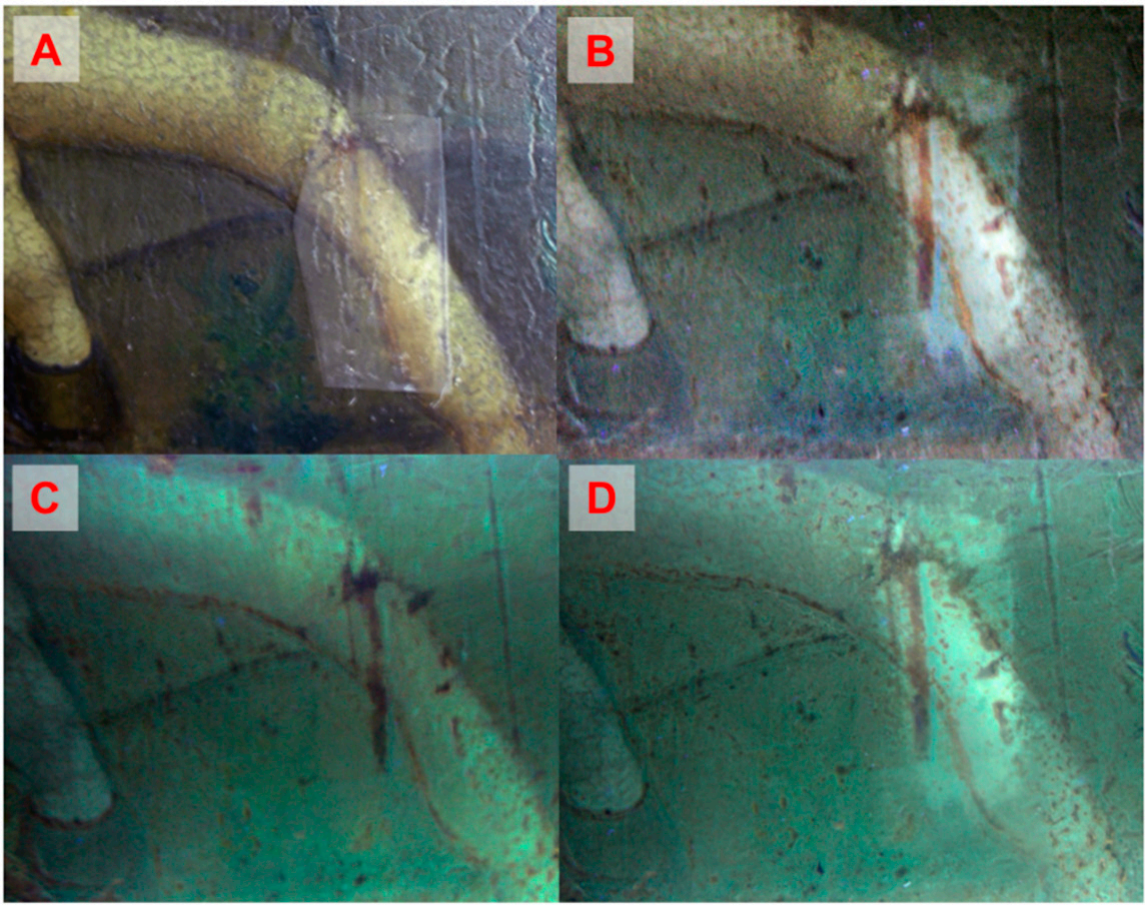
Soiled varnish removal from egg tempera paint with PE-PAMX_15_. Visible light reflectance images (**A**) during and (**B**) after treatment with the PE-PMAX_15_ loaded with a pH5 acetate aqueous solution without free surfactant. UV Fluorescence images (**C**) before and (**D**) after application. Image width corresponds to 3 cm.

The enhanced cleaning efficacy of the PE-PAM systems with incorporated surfactant is also demonstrated by removal of soiled mastic varnish from egg tempera early twentieth century panel painting. Figure 5 shows the cleaning action of a PE-PAMX_15_ hydrogel loaded with an aqueous pH5 acetate solution without free surfactant.

#### 2.5.3. Test 3—Surface Solubilization of Dammar Varnish

**Figure 6:**
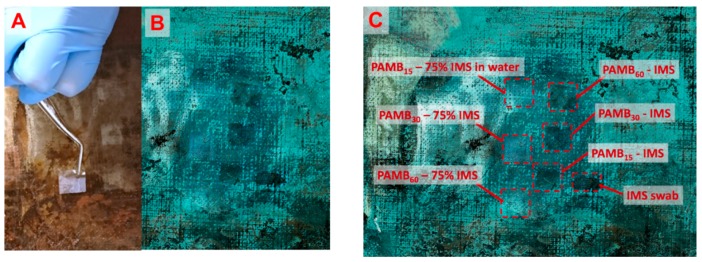
Demonstration of the cleaning treatment on a twentieth century oil painting with (**A**) application of the swollen PE-PAM sheet on the painting surface, (**B**) UV fluorescence overview of treaded areas, and (**C**) indication of experimental details for the specific treatment procedures. Darker image levels in the treated areas indicate more varnish removal. The PAM sheet dimension was 1 cm × 1 cm.

Surface layer solubilization of the dammar varnish of a twentieth century oil painting is demonstrated in Figure 6 with the PE-PAMB_60_, PE-PAMB_30_, and PE-PAMB_15_ systems swollen either with neat industrial methylated spirit (IMS, denatured alcohol) or as 75 %wt aqueous IMS solution. Prior to application, swab rolling pre-testing showed that 75 %wt IMS removed the varnish, but also affected the paint, as pigment traces were observed on the swab. 

The level of varnish removal was monitored by UV fluorescence imaging, exploiting the autofluorescence of the dammar varnish. In the swab cleaning test, the varnish was completely removed, as seen by the dark region indicated as "IMS swab" in Figure 6. Different fluorescence intensity levels indicated different levels of varnish removal in dependence of the PE-PAM type in combination with the solution composition and application conditions (which were kept the same for this test with a contact time of 10 s). The PE-PAMB_60_ system primed with 75 %wt IMS solution provided the most selective surface solubilization of the varnish with only a minute change in fluorescence. The largest change in fluorescence was found for the PE-PAMB_15_ loaded with the undiluted IMS that was selective, and yet, in contrast to the swab, it did not remove the entire varnish. 

These tests clearly document the high level of control over the cleaning procedure with thin PE-PAMB gels. The developed methodology is capable of instigating solubilization at varnish surfaces with minimum solution volume, which is restricted by the thin PAM network layer. We thus postulate that, in comparison with other established liquid treatment procedures (including swab- and gel-based methods), diffusion of the treatment solutions into the bulk of the painting layers is minimized by the architecture of the PE-PAM sheet. Similar results may only be possible by laser ablation [20,28,29]. 

## 3. Conclusions

We introduced the application of thin, surface-attached hydrogel layers for the surface treatment of painted works of art. Preliminary cleaning tests documented that selective removal of soils from varnish, complete varnish removal from paint, and controlled solubilization of the varnish surface itself are all possible with these ultrathin hydrogel film systems.

The specific characteristics of such hydrogel materials can be tailored by the following:

(a) polymer synthesis with full control over the comonomer composition, which can be expanded to other monomer types, and

(b) chemical functionalization of reactive groups in the polymer backbone.

In the present study, covalent incorporation of surfactant moieties via customized comonomers allowed tuning of the hydrophilic–hydrophobic balance of the hydrogel matrix, allowing surfactant-free cleaning solutions to be utilized and preventing surfactant leaching onto the artwork surface.

Furthermore, control over the degree of crosslinking by UV irradiation allowed to adjust the liquid capacity of the hydrogel layer. By tuning the crosslink density, sufficient softness can also be provided to the hydrogel, and in combination with the flexible support, conformal contact with painting surface is ensured.

From these promising application results, we envision potential extension to a wider variety of paints and coatings, and transfer of this technology to other conservation applications in cultural heritage.

Although the methodology was developed for the cleaning of paintings, with appropriate adaption of the hydrogel characteristics and swelling liquids, our technology may be well applicable to a wider scope of surface decontamination problems, as commonly found in the optical, electronics, and medical sectors.

## 4. Materials and Methods

### 4.1. Materials

Acrylamide (AM, 98%, Fluka, Buchs, Switzerland), glacial acetic acid (99.5%, Acros Organics, (Thermo Fischer), Geel, Belgium), and aqueous sodium hydroxide (1 M, Acros Organics) were used as received. The polyethoxylate surfactants included 4-(1,1,3,3-tetramethylbutyl)phenyl-polyethylene glycol (Triton X-100, Acros Organics), poly(oxyethylene)-23 lauryl ether (Brij 35, Sigma-Aldrich, St. Louis, MI, USA), and ethylene oxide-propylene oxide copolymer mono(2-ethylhexyl) ether (Ecosurf EH-3 (= EO3) or EH-9 (= EO9), Stockmeier Chemie GmbH & Co. KG, Bielefeld, Germany). Acryloyl chloride (96%, Alfa-Aesar, (Thermo Fischer), Heysham, UK), trimethylamine (TEA) (99%), and methacrylic acid (MAA) (99%, Sigma-Aldrich) were freshly distilled before use. Azobisisobutyronitrile (AIBN, 98%, Acros Organics) was recrystallized from methanol. Organic solvents, including xylene (Riedel & Haen, (Honeywell), Morristown, NJ, USA), ethyl acetate (EA, Sigma-Aldrich), ethanol (Merck, Darmstadt, Germany), and industrial methylated spirit (denaturated ethanol, IMS, Sigma-Aldrich), were used as received. Varnished substrates were made of polycyclohexanone (Laropal^®^ K80, BASF, Ludwigshafen, Germany) resins. Pure DI water of low conductivity (8 μS/cm) at pH 6.4 was utilized.

^1^H-NMR (Bruker AMX-300 spectrometer, Billerica, MA, USA) confirmed identity of the synthesized monomers and polymers.

### 4.2. Syntheses

#### 4.2.1. Monomers

*TXA* (4-(1,1,3,3-tetramethylbutyl)phenyl-polyethylene glycol acrylate): Triton X-100 acrylate was prepared according to the literature [55].

*B35A* (poly(oxyethylene) lauryl ether acrylate): Acryloyl chloride (1.66 mmol, 0.150 g) in dichloromethane (10 mL) was slowly added to a solution of Brij 35 (0.83 mmol, 1.000 g) and TEA (0.83 mmol, 0.116 mL) in dichloromethane (10 mL) at 0 °C under argon atmosphere. After stirring for 24 h at room temperature, the reaction mixture was washed with 5 wt% aqueous NaOH. The organic phase was dried with magnesium sulphate and removed under reduced pressure to yield 0.892 g (86%) of the product as white, waxy solid.

^1^H NMR (400 MHz, CDCl_3_), δ (ppm): 0.06 (s, 6.55 H), 0.87 (t, 3 H, CH_3_), 1.24 (s, 20 H, (CH_2_)_10_), 1.56 (quintet, 2 H, CH_2_), 1.92 (s, 1.19 H), 3.43 (t, 2 H, CH_2_), 3.56 (t, 2 H, CH_2_), 3.63 (s, 84 H, (C_2_H_4_O)_20_ + CH_2_), 3.72 (t, 2 H, CH_2_), 3.81 (m, 0.5 H), 4.32 (t, 2 H, CH_2_), 5.85–6.40 (m, 3 H, vinyl group).

*EO3A* (ethylene oxide-propylene oxide copolymer mono (2-ethylhexyl) ether acrylate): Acryloyl chloride (5.52 mmol, 0.450 g) in dichloromethane (20 mL) was slowly added to a solution of EO3 (2.76 mmol, 1.440 g) and TEA (2.76 mmol, 0.380 mL) in dichloromethane (20 mL) at 0 °C under argon atmosphere. After stirring for 24 h at room temperature, the reaction mixture was washed with 5 wt% aqueous NaOH. The organic phase was dried with magnesium sulphate and removed under reduced pressure to yield 1.49 g (93%) of the product as a viscous, slightly yellow liquid.

^1^H NMR (400 MHz, CDCl_3_), δ (ppm): 0.88 (t, 3 H, CH_3_), 0.90 (t, 3 H, CH_3_), 1.12 (m, 4 H, 2 CH_2_), 1.27 (m, 1 H, CH + 2 H, CH_2_), 1.49 (m, 2 H, CH_2_), 3.35–3.52 (m, CH_2_ + CH), 4.27 (t, 2 H, CH_2_), 5.8–6.4 (m, 3 H, vinyl H).

*EO9A* (ethylene oxide-propylene oxide copolymer mono (2-ethylhexyl) ether acrylate) was synthesized in the same way as EO3A, using EO9 instead of EO3 yielding 2.00 g (86%) of a viscous, slightly yellow liquid.

^1^H NMR (400 MHz, CDCl_3_), δ (ppm): 0.88 (t, 3 H, CH_3_), 0.90 (t, 3 H, CH_3_), 1.12 (m, 4 H, 2 CH_2_), 1.27 (m, 1 H, CH + 2 H, CH_2_), 1.49 (m, 2 H, CH_2_), 3.35–3.52 (m, CH_2_ + CH), 4.27 (t, 2 H, CH_2_), 5.8–6.4 (m, 3 H, vinyl H).

The methacrylic derivatives of Triton X-100 (TXM) and Brij 35 (B35M) were synthesized in analogy to their acrylic counterparts by using methacryloyl chloride instead of acryloyl chloride.

*TXM* (4-(1,1,3,3-tetramethylbutyl)phenyl-polyethylene glycol methacrylate): yield 0.920 g (78%) of a viscous, slightly yellow liquid.

^1^H NMR (400 MHz, CDCl_3_), δ (ppm): 0.69 (s, 9 H, (CH_3_)_3_), 1.32 (s, 6 H, (CH_3_)_2_), 1.68 (s, 2 H, CH_2_), 3.61–3.72 (m, 33 H, (C_2_H_4_O)_n_), 3.83 (t, 2 H, CH_2_), 4.09 (t, 2 H, CH_2_), 4.30 (t, 2H, CH_2_), 5.83–6.39 (m, 3 H, vinyl H), 6.81 (d, 2 H, aryl H), 7.22 (d, 2 H, aryl H).

*B35M* (poly(oxyethylene) lauryl ether methacrylate): yield 0.912 g (87%) of a white, waxy solid

^1^H NMR (400 MHz, acetonitrile), δ (ppm): 0.88 (t, 3 H, CH_3_), 1.27 (s, 20 H, (CH_2_)_10_), 1.52 (m, 2 H, CH_2_), 1.92 (t, 3 H, CH_3_), 2.16 (s, 8 H, H_2_O), 3.40 (t, 2 H, CH_2_), 3.50 (t, 2 H, CH_2_), 3.55 (s, 88 H, (C_2_H_4_O)_21_ + CH_2_), 3.68 (t, 2 H, CH_2_), 4.23 (t, 2 H, CH_2_), 5.63–6.06 (m, 2 H, vinyl group).

*BPAAm* (*N*-(4-benzoylphenyl)acrylamide) was synthesized from 4-aminobenzophenone and acryloyl chloride in analogy to the literature using sodium carbonate instead of TEA [56].

#### 4.2.2. Polymers

*PAM*: The photocrosslinkable polyacrylamide (PAM) copolymer with 99 mole equiv. acrylamide (AM) and 1 mole equiv. BPAAm was synthesized according to the literature [57].

##### PAMX

AM_94_/TXA_5_/BPAAm_1_: A mixture of AM (4.136 mmol, 0.294 g, 94 mole equiv.), TXA (0.220 mmol, 0.150 g, 5 mole equiv.), BPAAm (0.044 mmol, 0.011 g, 1 mole equiv.), and AIBN (0.022 mmol, 0.004 g) in dry dioxane (15 mL) was purged with argon for 15 min and stirred for 24 h at 65 °C. The product was precipitated in ethyl acetate (EA, five-fold volume of reaction mixture), separated from the solvent by centrifugation, and dried overnight under reduced pressure to yield 0.271 g (60%) of a white powder.

^1^H NMR (400 MHz, d6-DMSO + 1 droplet of D_2_O), δ (ppm): 0.67 (s, 9 H, (CH_3_)_3_), 1.24 (t, CH_2_CH_3_, EA), 1.55 (s, (CH_3_)_2_), 1.4–1.9 (m, 66 H, CH_2_–backbone), 1.90 (s, 1 H, CH_2_), 2.07 (s, CH_3_CO, EA), 2.15–2.5 (m, 34 H, CH–backbone), 2.71 (s, (CH_3_)_2_, DMSO), 3.45–3.70 (m, 25 H, (C_2_H_4_O)_n_), 3.75 (s, CH_2_, Dioxane), 4.12 (q, CH_2_CH_3_, EA), 4.79 (s, H_2_O), 5.80–6.23 (m, 2 H, monomer double bond), 6.77–7.15 (m, 4 H, aromatic H), 7.5–8 (m, 5 H, aromatic H).

AM_94_/TXM_5_/BPAAm_1_ was synthesized in analogy to AM_94_/TXA_5_/BPAAm_1_ using TXM instead of TXA. The product was obtained as 0.936 g (80%) of a white powder.

^1^H NMR (400 MHz, d6-DMSO + 1 droplet of D_2_O), δ (ppm): 0.67 (s, 9 H, (CH_3_)_3_), 0.85 (m, 4 H, (CH_3_)_2_),1.03 (m, 1.5 H, CH_3_), 1.16 (s, EA), 1.24, 1.29, 1.3–1.8 (m, 50 H, CH_2_–backbone and CH_2_ (TX100M)), 1.9–2.45 (m, 32 H, CH–backbone), 2.5 (DMSO), 3.35 (s, H_2_O), 3.4–3.65 (m, 34 H, (C_2_H_4_O)_n_), 3.56 (s, CH_2_, Dioxane), 4.03 (q, CH_2_CH_3_, EA), 3.72, 5.60–6.23 (m, 1.5 H, monomer double bond), 6.3–7.5 (m, aromatic H and amide H), 7.5–7.9 (m, 5 H, aromatic H).

##### PAMB

AM_94_/B35A_5_/BPAAm_1_: A mixture of AM (6.26 mmol, 0.445 g, 94 mole equiv.), B35A (0.333 mmol, 0.400 g, 5 mole equiv.), BPAAm (0.066 mmol, 0.016 g, 1 mole equiv.), and AIBN (0.033 mmol, 0.005 g) in dry dioxane (15 mL) was purged with argon for 15 min and stirred for 24 h at 65 °C. The product was isolated as stated for PAMX with a yield of 0.717 g (83%) as white powder.

^1^H NMR (400 MHz, d6-DMSO + 1 droplet of D_2_O), δ (ppm): 0.83 (t, 3 H, CH_3_), 1.21 (m, 22 H, (CH_2_)_11_), 1.4–1.6 (m, 52 H, CH_2_–backbone (+B35A)), 1.9–2.1 (m, 37 H, CH–backbone (+B35A)), 2.48 (DMSO), 3.48 (s, 44 H, (C_2_H_4_O)_13_), 3.55 (s, CH_2_, dioxane), 3.62 (s, 53 H, H_2_O associated to polymer chain), 5.67–6.15 (m, monomer double bond), 6.45–7.5 (m, 55 H, amide H ), 7.5–7.7 (m, 6 H, aromatic H).

AM_94_/B35M_5_/BPAAm_1_ was synthesized in analogy to AM_94_/B35A_5_/BPAAm_1_ using B35M instead of B35A, yielding 0.562 g (67%) of a white powder.

^1^H NMR (400 MHz,), δ (ppm): 0.85 (t, 3 H, CH_3_), 1.05 (m, CH_3_), 1.16, 1.23 (s, (CH_2_)_n_), 1.3–1.8 (m, 14 H, CH_2_–backbone (+B35M)), 1.8–2.45 (m, 10 H, CH–backbone (+B35M)), 2.50 (DMSO), 3.34 (s, H_2_O), 3.50 (s, 21 H, (C_2_H_4_O)_n_), 3.56 (s, dioxane), 4.04, 4.32, 5.60–6.15 (m, 1.5 H, monomer double bond), 6.5–7.5 (m, 17 H, amide H ), 7.5–7.72 (m, 2 H, aromatic H).

AM_89_/B35A_5_/MAA_5_/BPAAm_1_: AM (5.927 mmol, 0.421 g), B35A (0.333 mmol, 0.400 g), BPAAm (0.066 mmol, 0.016 g), and AIBN (0.061 mmol, 0.010 g) were dissolved in dry dioxane (10 mL) and purged with argon for 15 min. Then, MAA (0.333 mmol, 0.028 mL) was added and the mixture stirred for 40 h at 65 °C. Product isolation was performed as stated above for PAMX.

^1^H NMR (400 MHz, d6-DMSO), δ (ppm): 0.85 (t, CH_3_), 1.00 (s, CH_3_), 1.21 (m, (CH_2_)_n_), 1.3–1.8 (m, CH_2_–backbone (+B35A)), 1.8–2.4 (m, CH–backbone (+B35A)), 2.50 (DMSO), 3.34 (H_2_O), 3.50 (s, (C_2_H_4_O)_n_), 3.56, 4.03–4.32, 5.60–6.20 (m, monomer double bond), 6.45–7.5 (m, amide H ), 7.5–7.9 (m, aromatic H), 12.13 (s, -COO*H*).

##### PAM-EO3 and PAM-EO9:

AM_94_/EO3A_5_/BPAAm_1_ (PAM-EO3): A mixture of AM (70.000 mmol, 5.000 g, 94 mole equiv.), EO3A (3.720 mmol, 2.150 g, 5 mole equiv.), BPAAm (0.750 mmol, 0.190 g, 1 mole equiv.), and AIBN (0.370 mmol, 0.061 g) in dry dioxane (50 mL) was purged with argon for 30 min and stirred for 24 h at 65 °C. The product was precipitated in ethyl acetate (five-fold volume of reaction mixture), separated from the solvent by centrifugation, and dried overnight under reduced pressure to yield 6.91 g (88%) of a white powder.

^1^H NMR (400 MHz, d6-DMSO), δ (ppm): 0.80 (s, 6 H, (CH_3_)_3_), 1.00 (t, 14 H, CH_3_), 1.21 (s, 18 H, CH_2_ + CH), 1.40–1.80 (m, 75 H, CH_2_-backbone), 2.07–2.40 (m, 40 H, CH-backbone), 2.50 (DMSO), 3.25 (m, 2 H, CH_2_), 3.29 (H_2_O), 3.37 (m, 4 H, CH–O), 3.48 (m, 13 H, CH_2_–O), 3.54 (s, dioxane), 6.54–7.40 (m, 75 H, amide H), 7.50–7.80 (m, 5 H, aromatic H).

AM_94_/EO-9A_5_/BPAAm_1_ (PAM-EO9) was synthesized in the same way as PAM-EO3 using EO-9A instead of EO-3A yielding 6.80 g (81%) of a white powder.

^1^H NMR (400 MHz, d6-DMSO), δ (ppm): 0.81 (s, 6 H, (CH_3_)_3_), 1.01 (t, 13 H, CH_3_), 1.22 (s, 10 H, CH_2_ + CH), 1.40–1.80 (m, 84 H, CH_2_-backbone), 2.07–2.40 (m, 53 H, CH-backbone), 2.50 (DMSO), 3.24 (m, 2 H, CH_2_), 3.32 (s, 4 H, CH_2_), 3.37 (m, 35 H, CH–O), 3.40 (s, 4H, CH–O), 3.49 (m, 19 H, CH_2_–O), 3.54 (s, dioxane), 6.84–7.40 (m, 85 H, amide H), 7.50–7.80 (m, 6 H, aromatic H).

##### PE-PAM Sheet Preparation

Food-quality storage bags were used as a source for the PE supports. After cutting, the sheets were fixed on a glass plate, cleaned with an ethanol-soaked tissue, and dried. Corona treatment was performed with a discharge device (Sicatech uni-systems lf1), operated at approximately 7kV output, 400 Watt. PE sheets were placed 2.5 cm below the discharger and moved slowly and continuously until the whole surface was treated. The sample contact time with the corona discharge was 2 s/10 cm^2^. 

PAM, PAMB, PAMX, PAM-EO3, or PAM-EO9 were dissolved (10 g/L) in H_2_O/EtOH (1:1 *v*/*v*). Then, 0.5 mL of this polymer solutions was casted on the prepared PE supports by doctor blading (1000 µm layer thickness when wet) and dried overnight. The samples were crosslinked under N_2_ atmosphere for 10, 15, 20, 30, or 60 min at 365 nm, corresponding to a UV energy dose of approximately 0.2–1 J/cm^2^. Afterwards, the sheets were immersed into water for 30 min to rinse non-crosslinked polymer fractions and dried overnight. Table 2 provides a list of the prepared sheet types along with the PE-PAM sample abbreviations. 

As the methacrylate derivatives did not show sufficient solubility and wetting of the PE supports, they were not used for the preparation of the PE-PAM sheets.

### 4.3. Stability Tests of PE-PAM Sheets

Plain PE (for reference), PE-PAM_15_ and PE-PAMB_15_ sheets with a size of 1.9 × 7.6 cm^2^ were fixed by clamping on a sample holder. A detailed image of the experimental setup is provided in the Supporting Information, Figure S15. Then, adhesive tape (Tesa) was applied on the center of the sheet by pressing from above with a glass plate (applied area: 1.9 × 1.9 cm^2^). For the force measurement, the adhesive tape was pulled off the foil with a constant speed of 1.5 mm/min using a Zwick 1425 tensile testing machine (Zwick/Roell, Ulm, Germany). The applied force was measured with a spring balance. 

### 4.4. Water Loading Test

Samples for these tests were cut out from the different PE-PAM sheets with either a sharp knife (sample size 19 × 76 mm) or a circular trephine with a diameter of 18 mm.

A Mettler Toledo AX105 Delta Range balance with a resolution of 0.01 mg was employed for the gravimetric analysis. Microscope slides (19 × 76 mm), which were used for sample protection during the weighing, were cleaned with soap, water, and ethanol and dried before usage. The samples were weighed dry, then immersed into water for 5 min, weighed in the swollen state, and dried overnight under reduced pressure at 40 °C before the last weighing (dry). In the swollen state, excess water was removed carefully from the sheet surface with a tissue before weighing.

### 4.5. Preliminary Cleaning Tests of Painting Surfaces with PE-PAM Sheets

#### 4.5.1. Soil Removal from Mock-Up Laropal K80 Varnish

On soiled and accelerated aged Laropal K80 varnish [58], swab rolling pre-tests were performed to confirm efficacy of the cleaning solution with following composition: 0.6% *v*/*v* Triton X-100 in 0.5% *v*/*v* CH_3_COOH buffered with 1 M NaOH to pH 5. Then, the PE-PAM_60_, PE-PAM_30_, and PE-PAM_15_ sheets were cut into pieces with dimensions of 1 cm^2^ and immersed into the cleaning solution to swell the active hydrogel layer. The PE-PAM sheets were handled with tweezers in all process steps. Excess of liquid was removed by blotting the PE support side on tissue paper. Then, the hydrogel side was brought in contact with the mock-up surface. To ensure sufficient contact with the mock-up, a soft cotton swab was gently rolled on the PE backing for less than 2 s. After an incubation time of 15 s, the PE-PAM sheets were removed and the surface left for 1 min to dry. Then, the surface was brought in contact for 10 s with a new PE-PAM sheet immersed in rinsing solution composed of 0.5% *v*/*v* CH_3_COOH buffered with 1M NaOH to pH 5 (no free surfactant) for clearance of free surfactant residues. Then, the cleaning efficacy of PE-PAMB sheets swollen with the rinsing solution was tested following exactly the same application methodology.

#### 4.5.2. Removal of Mastic Varnish from Egg Tempera Paint

A similar procedure as described above for the mock-up tests was performed on an early twentieth century egg tempera panel painting with a mastic varnish as a one-step approach, using only a PE-PAMX_15_ sheet with only the acetate buffer rinsing solution without free surfactant.

#### 4.5.3. Surface Solubilization of Dammar Varnish

As a pre-test, solutions of neat IMS and 75 %wt IMS in DI water were swab rolled to solubilize the dammar varnish of a twentieth century oil easel painting. Then, PE-PAMB_60_, PE-PAMB_30_, and PE-PAMB_15_ sheets were cut into pieces with dimensions of 1 cm^2^ and immersed in each solution, respectively. After the excess of solution was removed, the hydrogels were brought into contact with the varnish surface. Sufficient contact was obtained by swab rolling on the PE backing for less than 2 s, followed by incubation for 10 s.

## Figures and Tables

**Table 1:** Solubility of synthesized copolymers. Indicators explained: “+” soluble, “o” not completely soluble, “-” insoluble, “/” not tested; two signs indicate tendencies.

Copolymer Composition	H_2_O	EtOH	H_2_O: EtOH (1:1)	MeOH	Acetone	Diethyl Ether	EA	DMSO
AM_94_/B35A_5_/BPAAm_1_	o	-	o+	-	o -	-	-	+
AM_94_/TX100A_5_/BPAAm_1_	o+	/	o+	-	o-	-	-	/
AM_94_/B35M_5_/BPAAm_1_	-	-	o-	-	o-	-	-	o+
AM_94_/TX100M_5_/BPAAm_1_	o-	-	o+	-	o-	-	-	o+
AM_89_/B35A_5_/MAA_5_/BPAAm_1_	-	-	o+	-	o	-	-	o+
AM_94_/EO-3A_5_/BPAAm_1_	o+	-	+	-	o	-	-	+
AM_94_/EO-9A_5_/BPAAm_1_	o+	-	+	-	o	-	-	+

**Table 2:** List of prepared polyethylene-supported polyacrylamide (PE-PAM) sheet types with corresponding copolymer composition, UV crosslinking time, and sample abbreviations.

Abbreviation	Polymer Composition	t_cr_/min
PE-PAM_15_	AM_99_/BPAAm_1_	15
PE-PAM_30_	AM_99_/BPAAm_1_	30
PE-PAM_60_	AM_99_/BPAAm_1_	60
PE-PAMB_10_	AM_94_/B35A_5_/BPAAm_1_	10
PE-PAMB_15_	AM_94_/B35A_5_/BPAAm_1_	15
PE-PAMB_20_	AM_94_/B35A_5_/BPAAm_1_	20
PE-PAMB_30_	AM_94_/B35A_5_/BPAAm_1_	30
PE-PAMB_60_	AM_94_/B35A_5_/BPAAm_1_	60
PE-PAMX_15_	AM_94_/TXA_5_/BPAAm_1_	15
PE-PAMX_30_	AM_94_/TXA_5_/BPAAm_1_	30
PE-PAMX_60_	AM_94_/TXA_5_/BPAAm_1_	60
PE-PAM-EO3_15_	AM_94_/EO-3A_5_/BPAAm_1_	15
PE-PAM-EO9_15_	AM_94_/EO-9A_5_/BPAAm_1_	15

## Supplementary Materials

Supplementary materials can be found at https://www.mdpi.com/2310-2861/6/1/1/s1.

## Abbreviations

AFM	atomic force microscopy
AIBN	azobisisobutyronitrile
B35A	Brij 35 acrylate
B35M	Brij 35 methacrylate
BPAAm	benzophenone acrylamide
DI	deionized
DMSO-D_6_	fully deuterated dimethyl sulfoxide
DMSO	dimethyl sulfoxide
EA	ethyl acetate
EO3A/EO9A	Ecosurf EH-3 or EH-9 acrylate
EtOH	ethanol
^1^H-NMR	proton nuclear magnetic resonance
IMS	industrial methylated spirit (denatured alcohol)
MeOH	methanol
PAM	polyacrylamide
PAM_15_	“15” indicates 15 min photocrosslinking time for polyacrylamide layer
PAMB	copolymer of AM, B35A and BPAAm
PAM-EO3/EO9	copolymer of AM, EO3 or EO9, respectively, and BPAAm
PAMX	copolymer of AM, TXA and BPAAm
PE	polyethylene
PE-PAM	polyethylene -supported polyacrylamide
TEA	triethylamine
TXA	4-(1,1,3,3-tetramethylbutyl)phenyl-polyethylene glycol (Triton X-100) acrylate
TXM	Triton X-100 methacrylate
UV	ultraviolet light
