# Supplementary material for: Polyolefin-Supported Hydrogels for Selective Cleaning Treatments of Paintings
Source: Gels. 2019 Dec 18;6(1):1. doi: 10.3390/gels6010001 (PMC7151125; doi:10.3390/gels6010001)
Supplement: Supplementary file 1 [file gels-06-00001-s001.pdf]

Supporting Information.

Polyolefin-supported hydrogels for selective cleaning treatments of paintings

Silvia Freese[1], Samar Diraoui[1], Anca Mateescu[2], Petra Frank[1], Charis Theodorakopoulos[3], Ulrich Jonas[1]**

1 Macromolecular Chemistry, Department Chemistry - Biology, University of Siegen, Adolf-Reichwein-Strasse 2, D-57076 Siegen, Germany

2 Continental Automotive Romania, Research and Development, Display Technology Department, Strada Siemens 1, 300704 Timisoara, Romania

3 Department of Arts, Science in Conservation of Fine Art, Northumbria University, Newcastle upon Tyne, NE1 8ST, UK

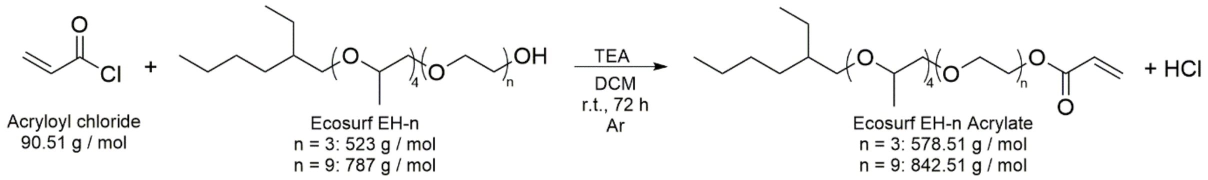

Figure S1: Synthetic pathways for the Ecosurf EH-n acrylates (EO-nA).

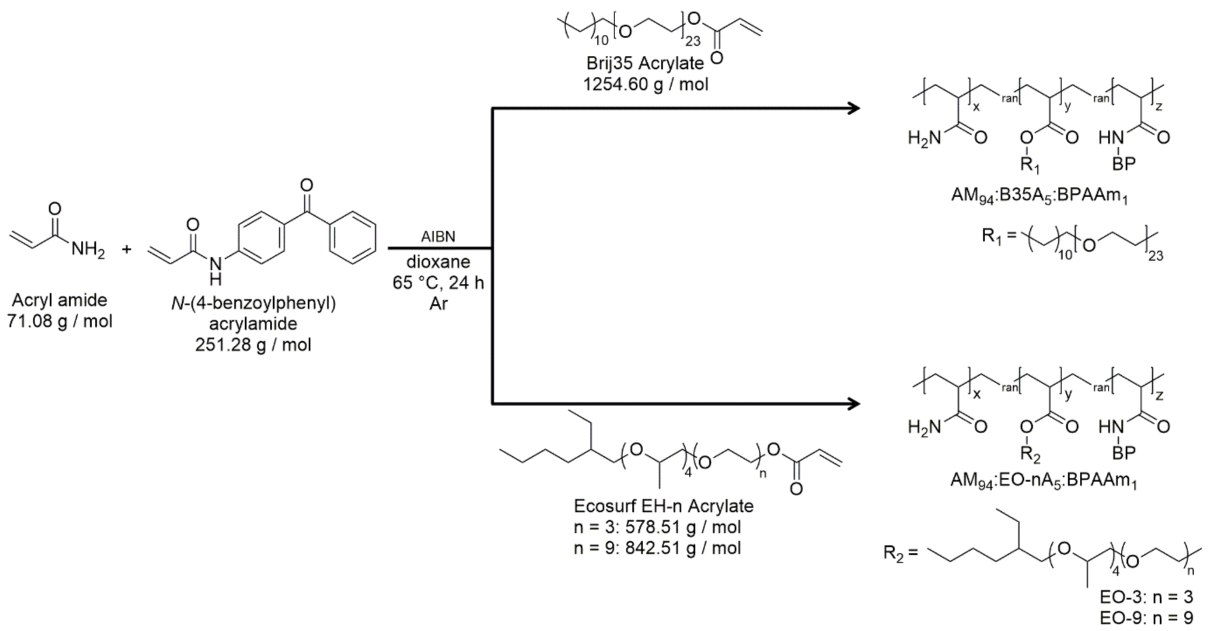

Figure S2: Synthesis scheme for the preparation of PAM copolymers with embedded Brij 35 or Ecosurf surfactant moieties.

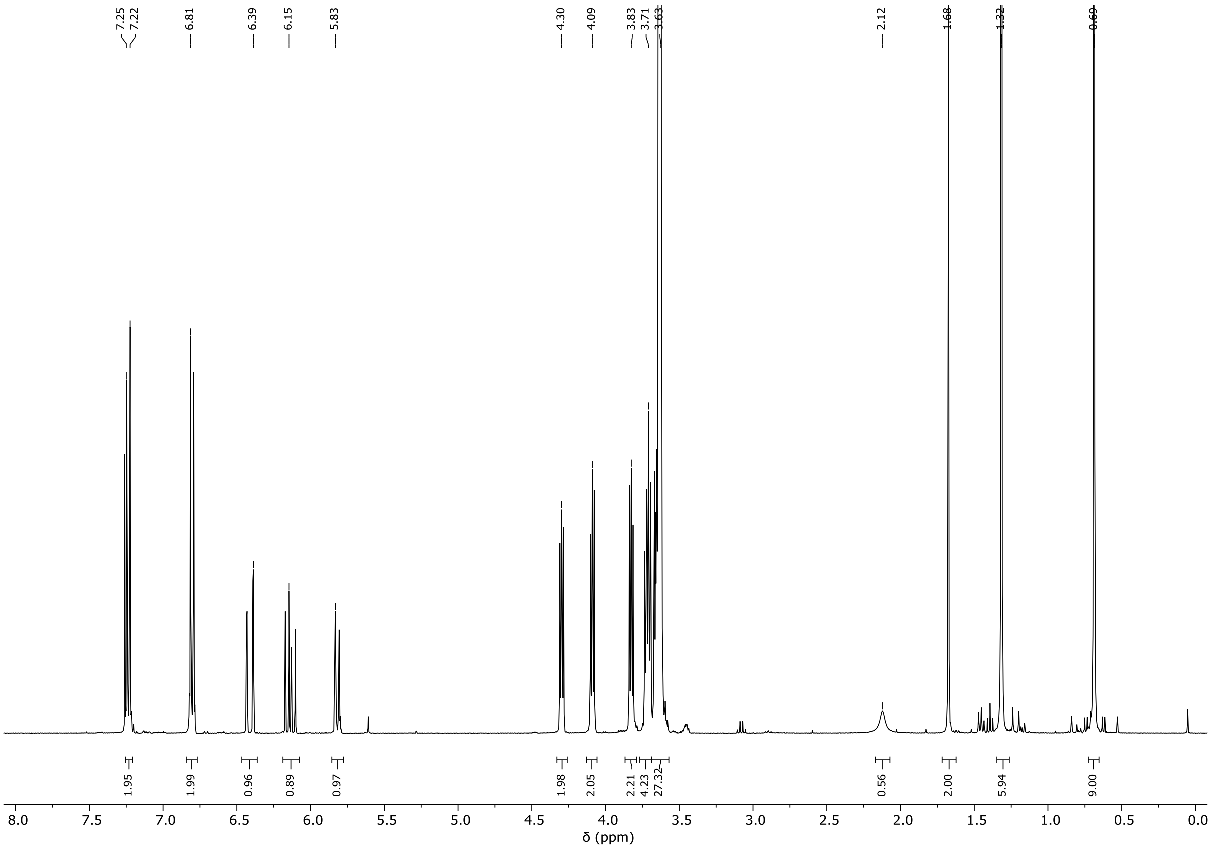

Figure S3: ^{1}H-NMR spectrum of TritonX100 acrylate (TXA) in $CDCl_3$.

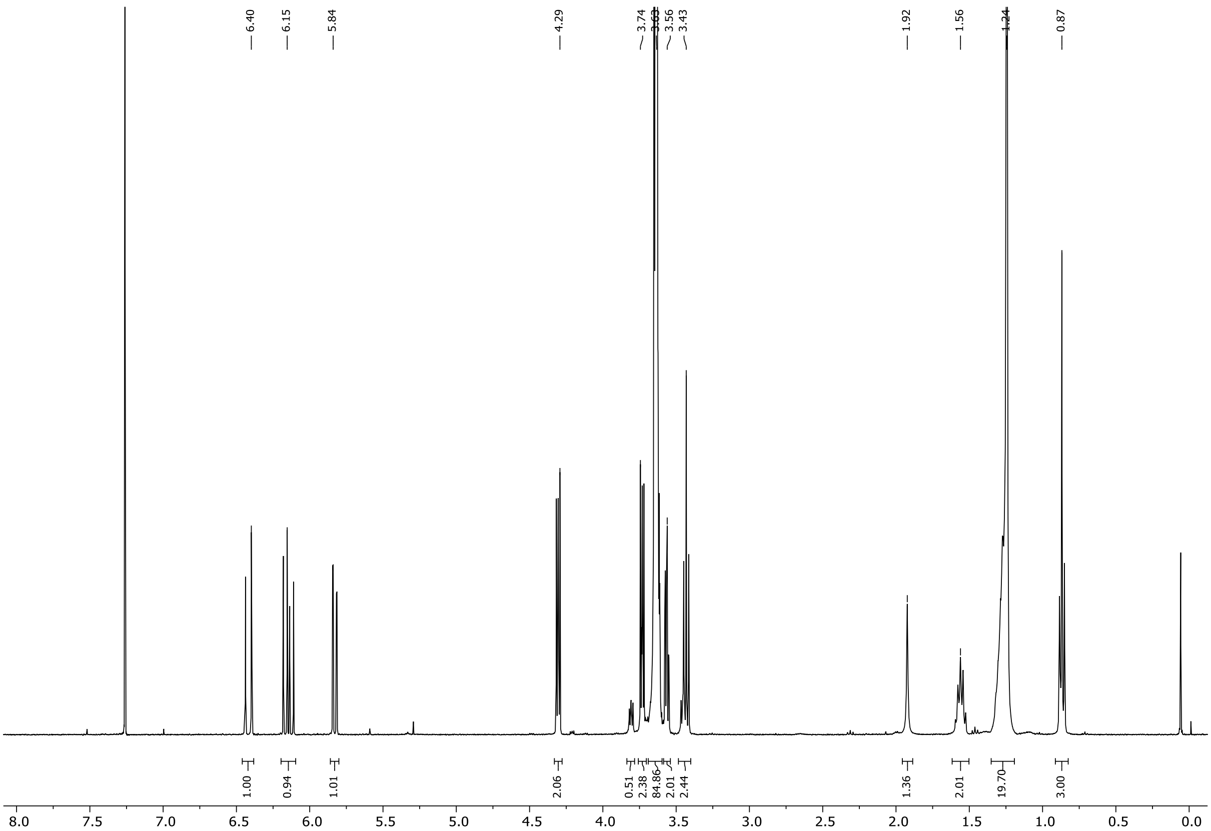

Figure S4: ^{1}H-NMR spectrum of Brij35 acrylate (B35A) in $CDCl_3$.

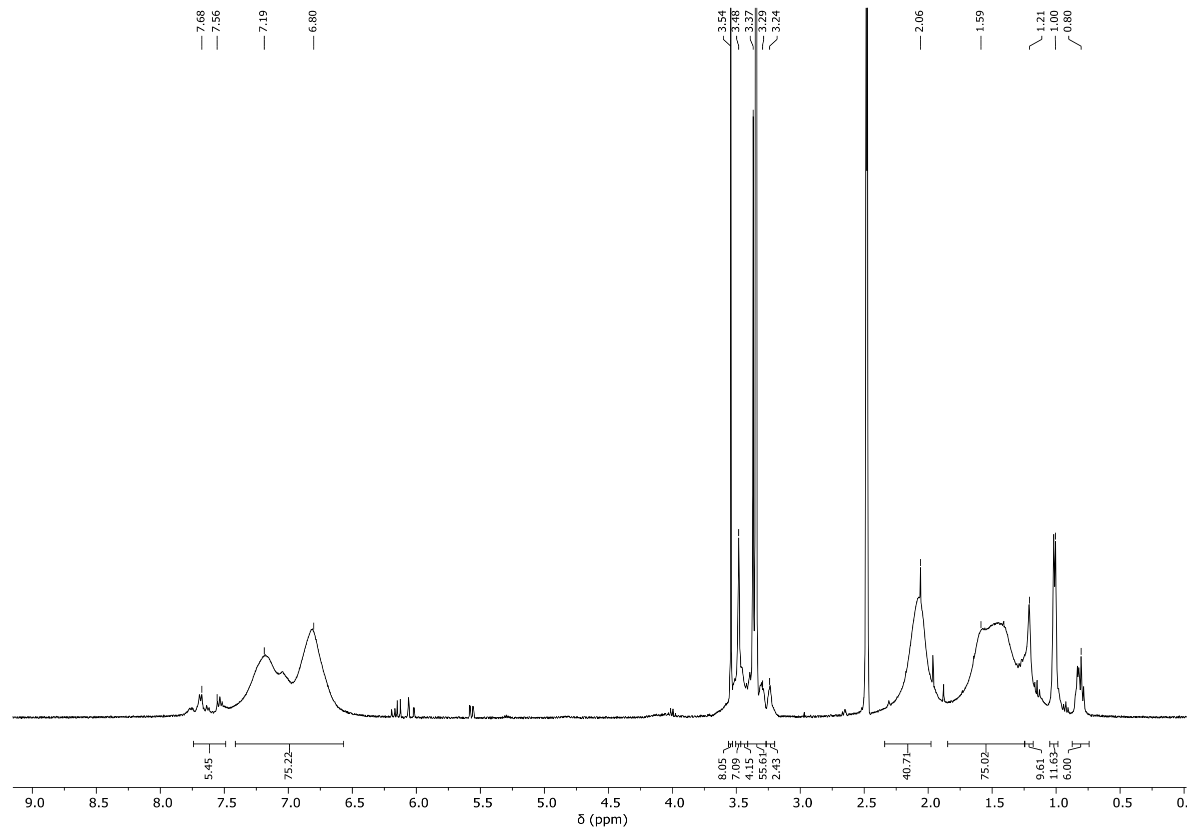

Figure S5: ^{1}H-NMR spectrum of Ecosurf EH-3 acrylate (EO-3A) in $CDCl_3$.

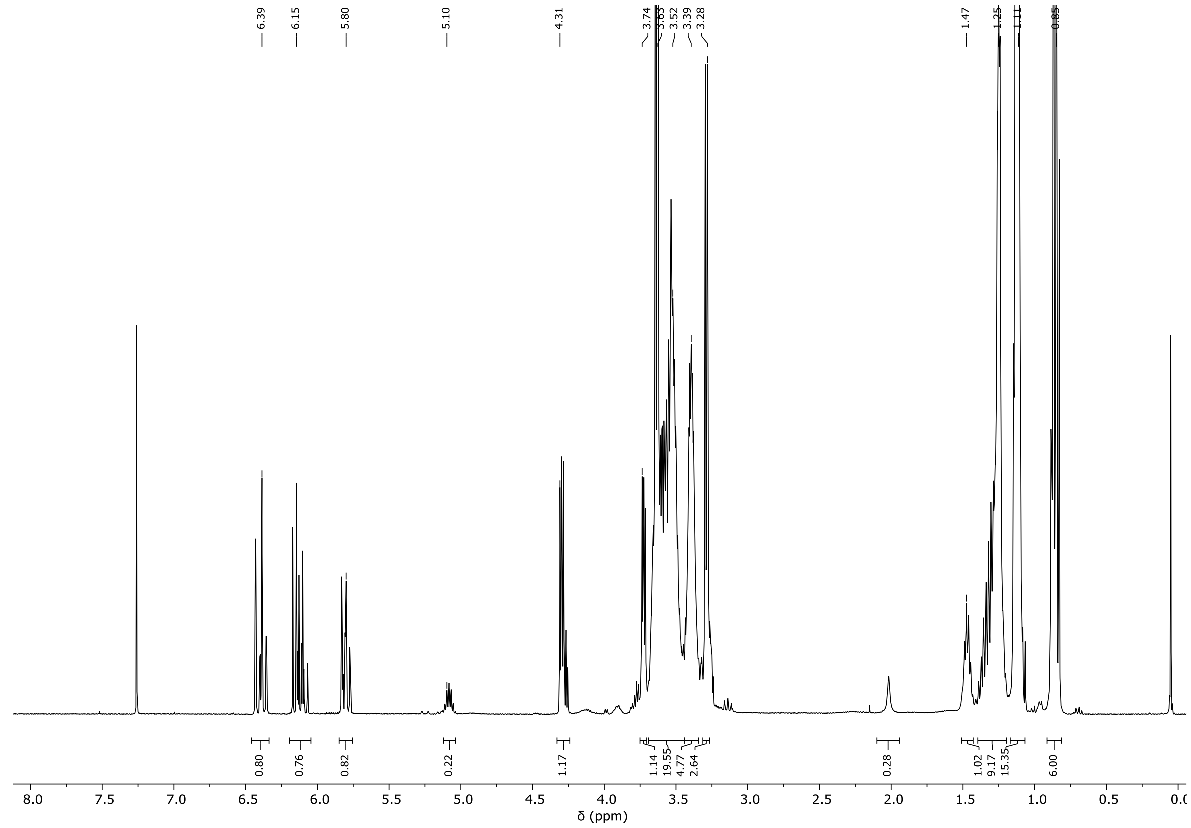

Figure S6: ^{1}H-NMR spectrum of Ecosurf EH-9 acrylate (EO-9A) in $CDCl_3$.

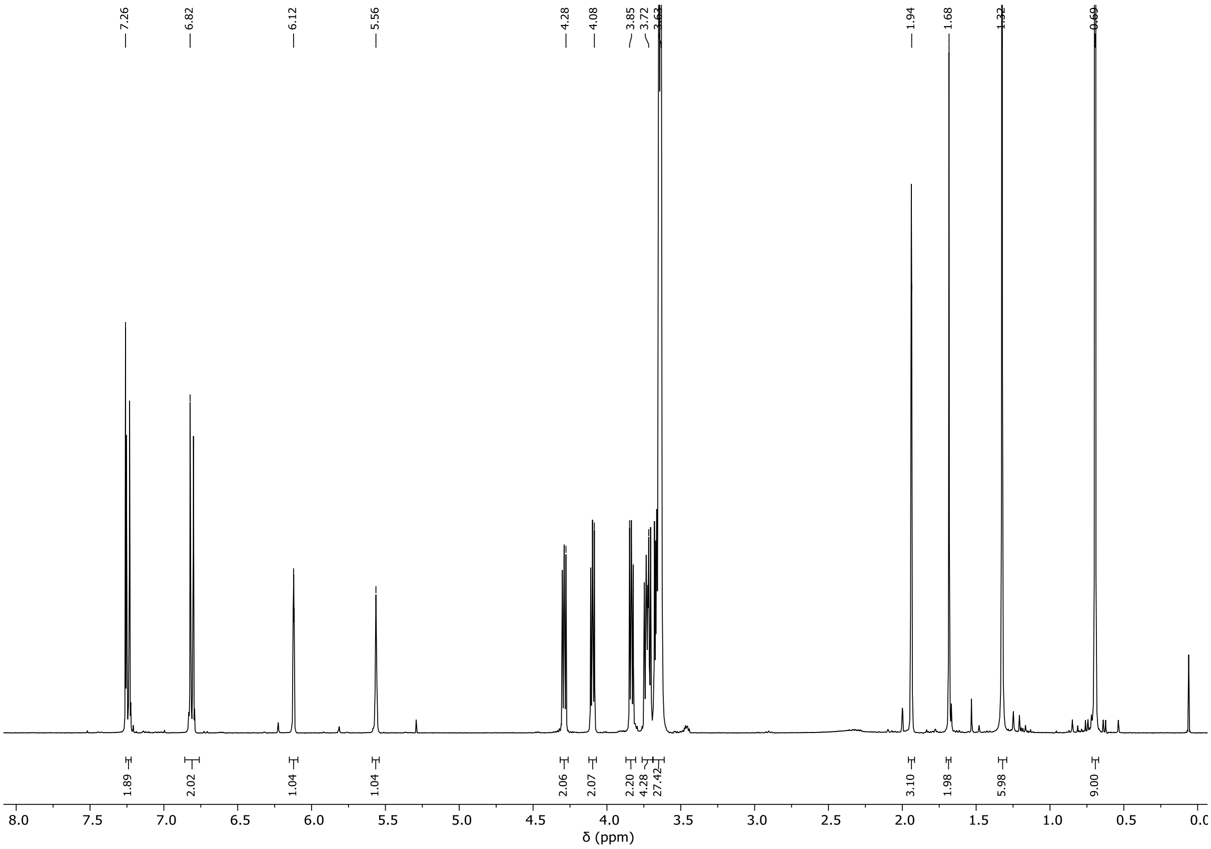

Figure S7: ^{1}H-NMR spectrum of TritonX100 methacrylate (TXM) in $CDCl_3$.

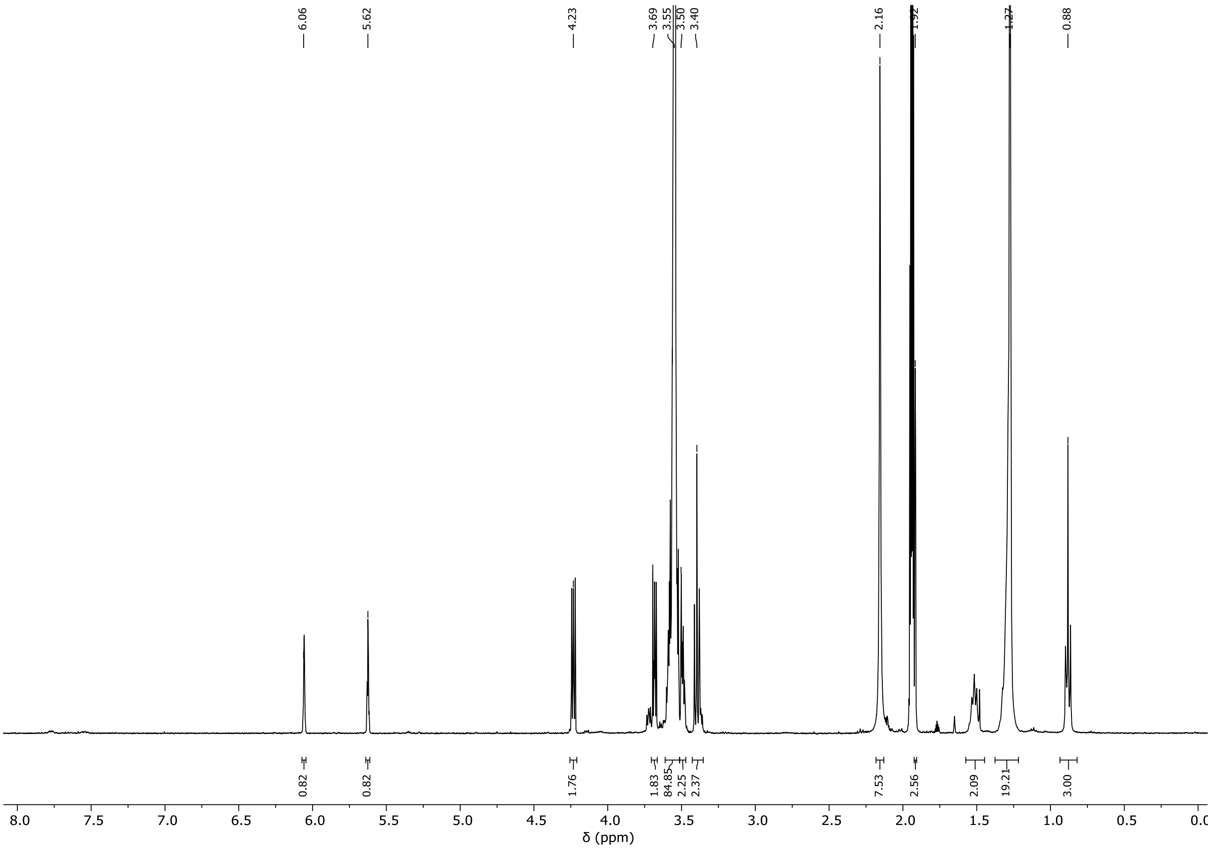

Figure S8: ^{1}H-NMR spectrum of Brij35 methacrylate (B35M) in acetonitrile-d3.

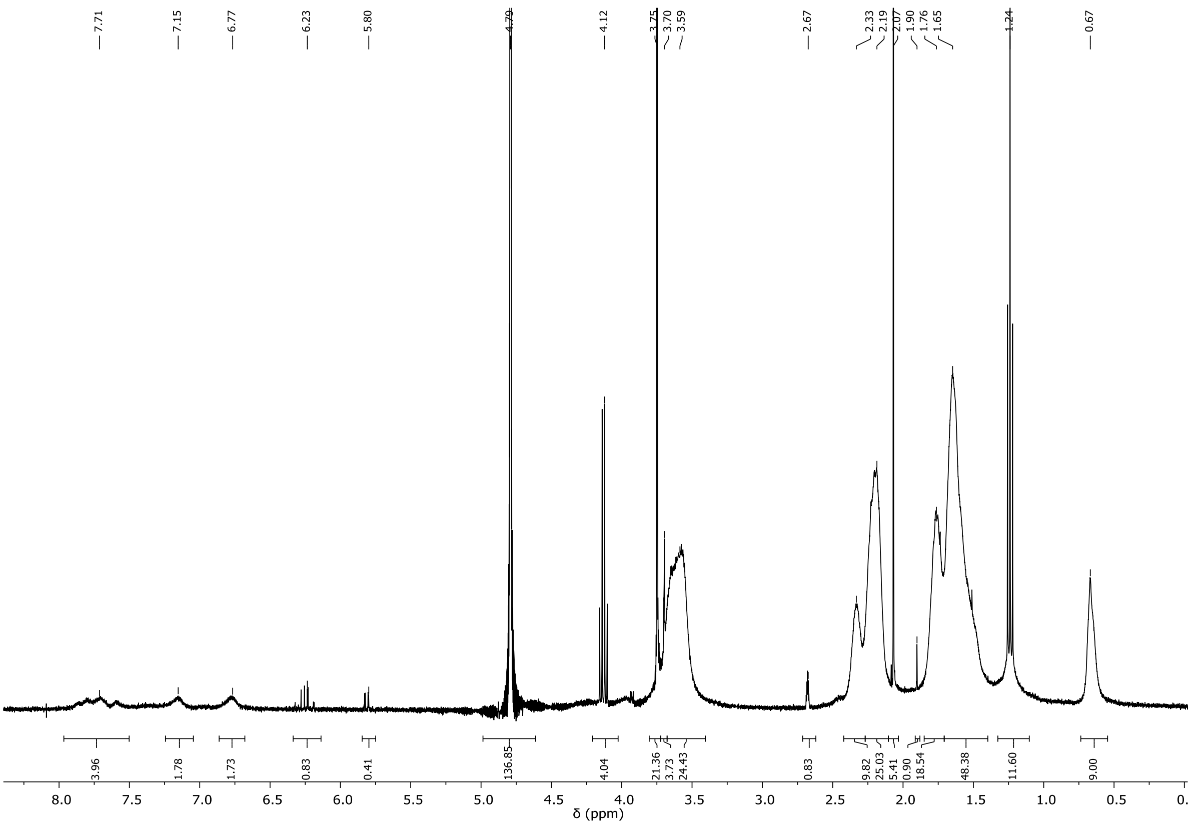

Figure S9: ^{1}H-NMR spectrum of PAMX terpolymer with a composition of AM_{94}:TXA_5:$BPAAm_1$ in DMSO-d_6 + 1 droplet of D_2O.

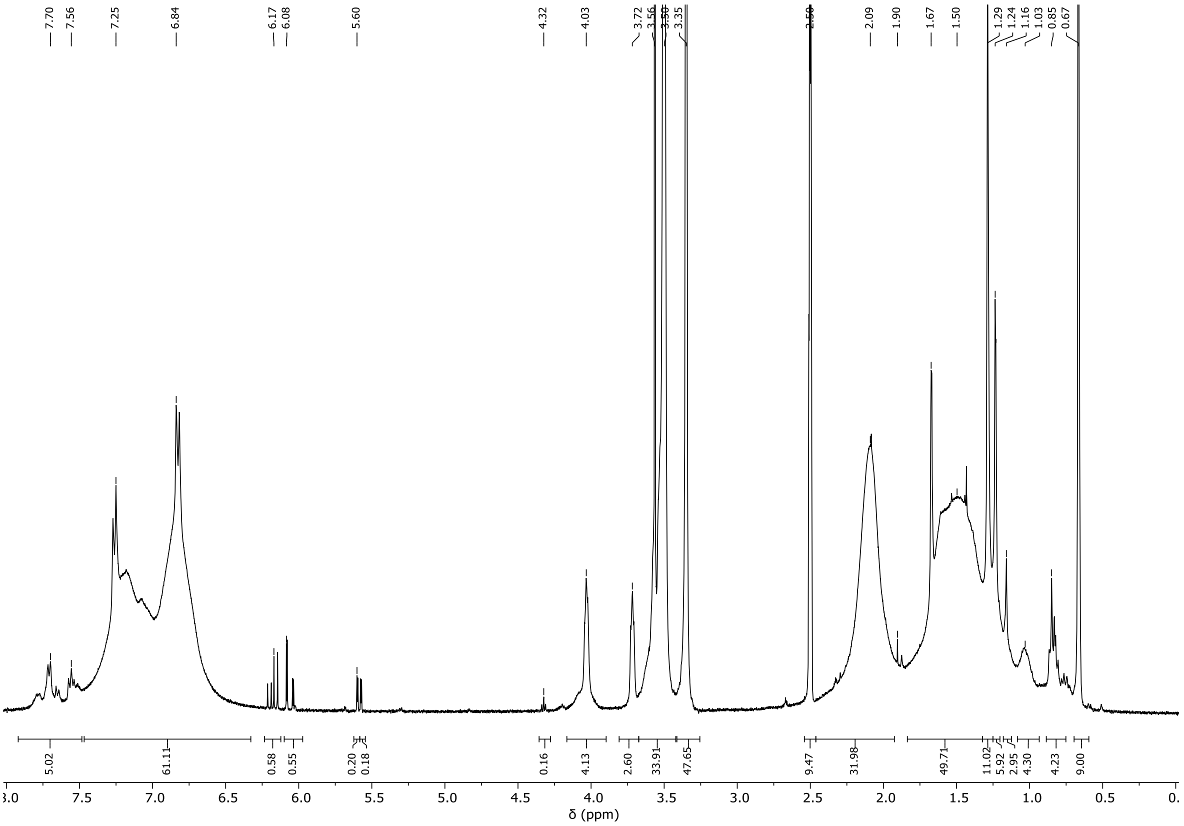

Figure S10: ^{1}H-NMR spectrum of PAMX methacrylate terpolymer with a composition of AM_{94}:TXM_5:$BPAAm_1$ in DMSO-d_6 + 1 droplet of D_2O.

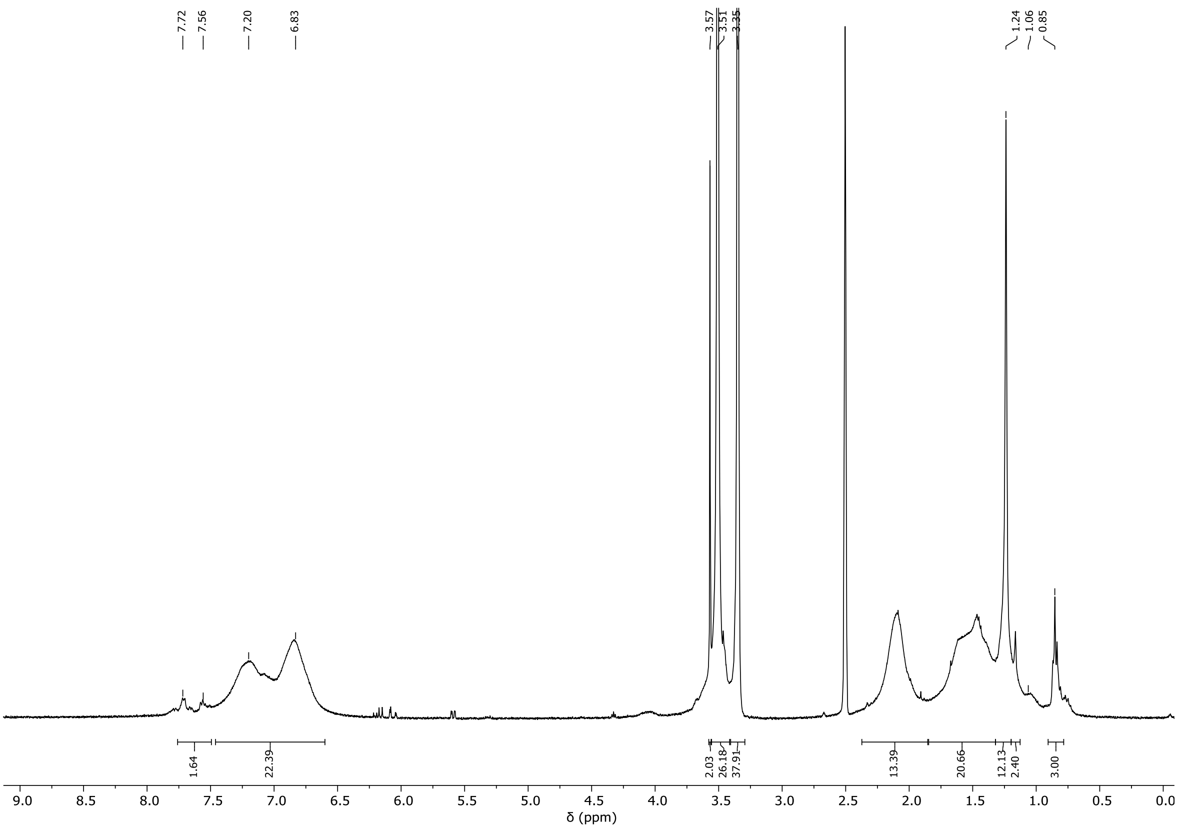

Figure S11: ^{1}H-NMR spectrum of the PAMB methacrylate terpolymer with a composition of AM_{94}:$B35M_5$:$BPAAm_1$ in DMSO-d_6

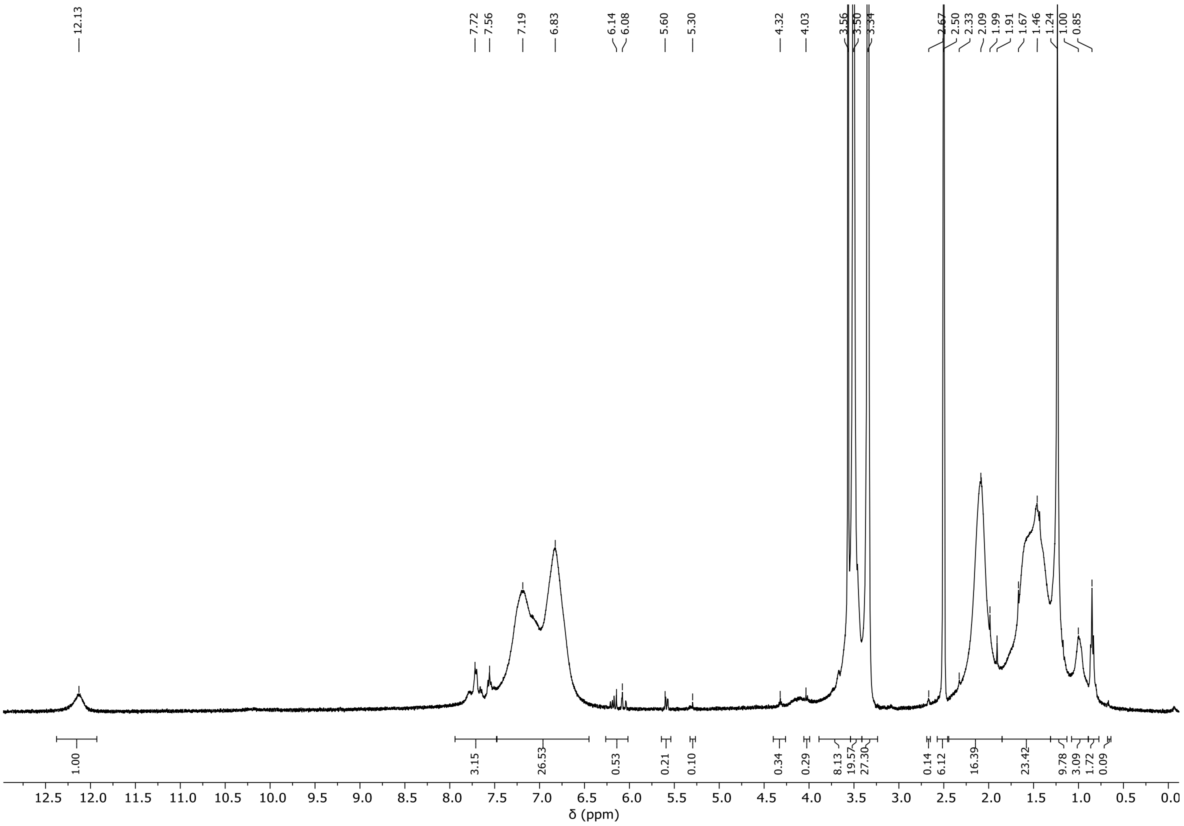

Figure S12: ^{1}H-NMR spectrum of the PAMB-MAA quadropolymer with a composition of AM_{89}:$B35A_5$:MAA_5:$BPAAm_1$ in DMSO-d_6

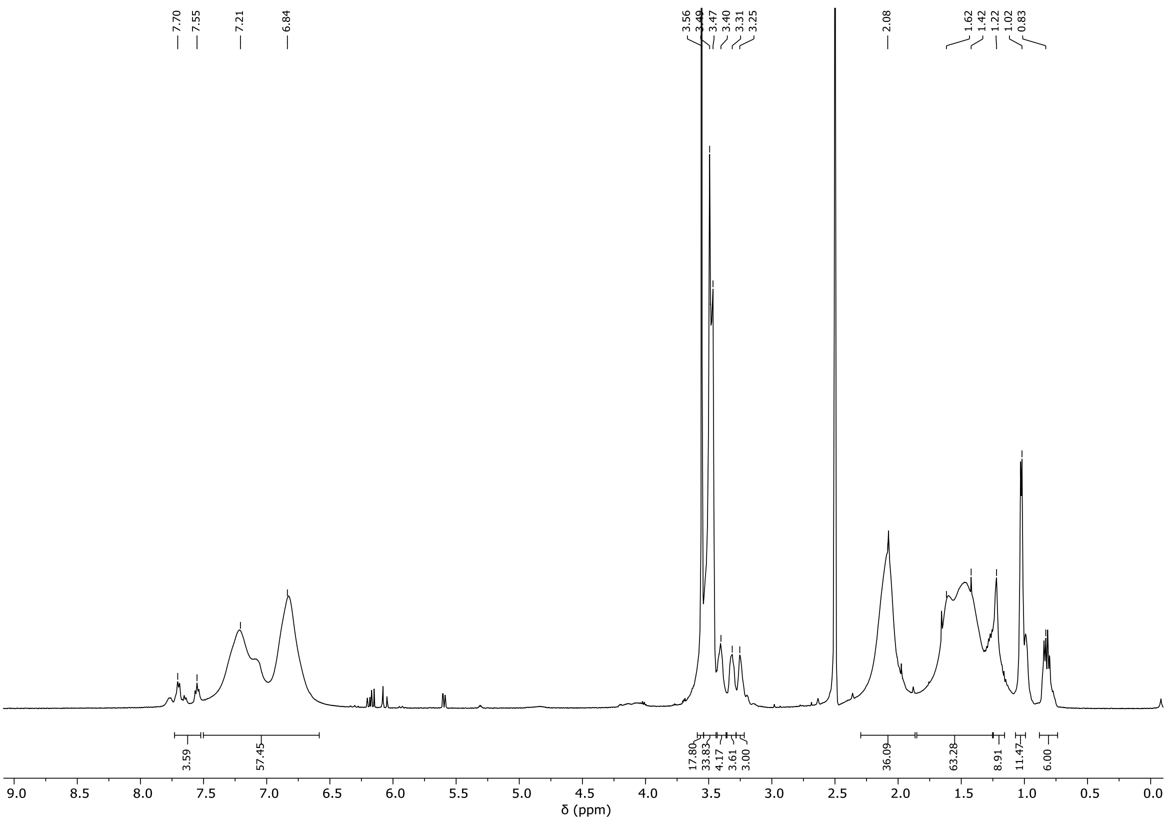

Figure S13: ^{1}H-NMR spectrum of the PAM-EO3 terpolymer with a composition of AM_{94}:$EOA3_5$:$BPAAm_1$ in DMSO-d_6

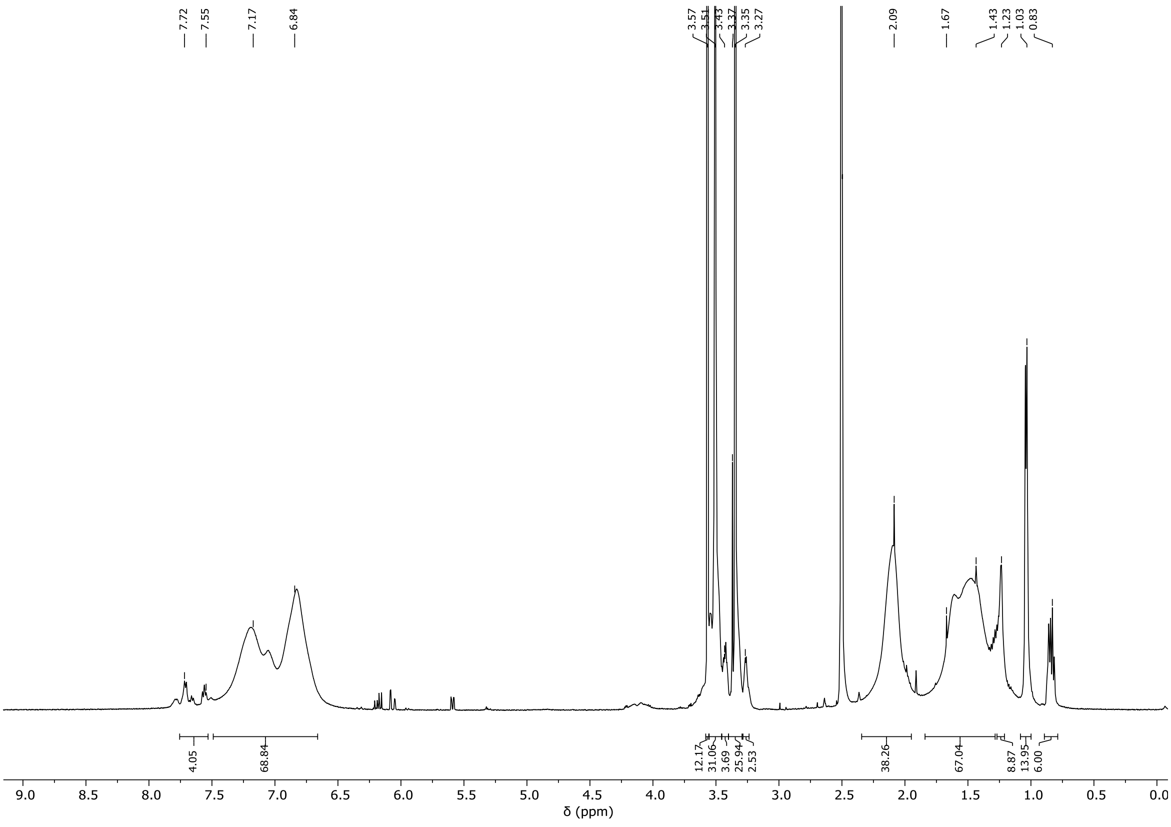

Figure S14: ^{1}H-NMR spectrum of the PAM-EO9 terpolymer with a composition of AM_{94}:$EOA9_5$:$BPAAm_1$ in DMSO-d_6

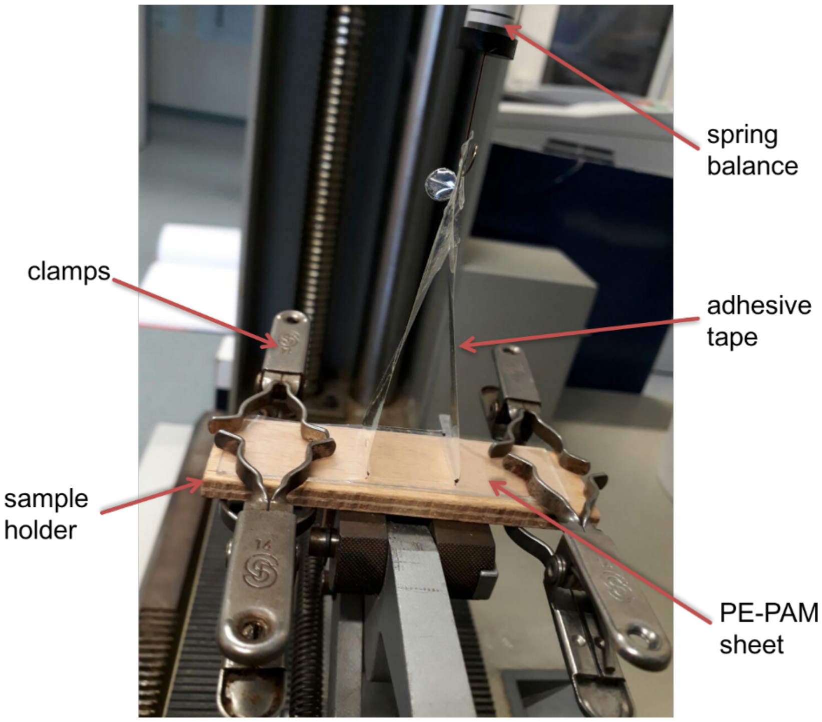

Figure S15: Setup for the stability tests of the PE-PAM sheet with adhesive tape.

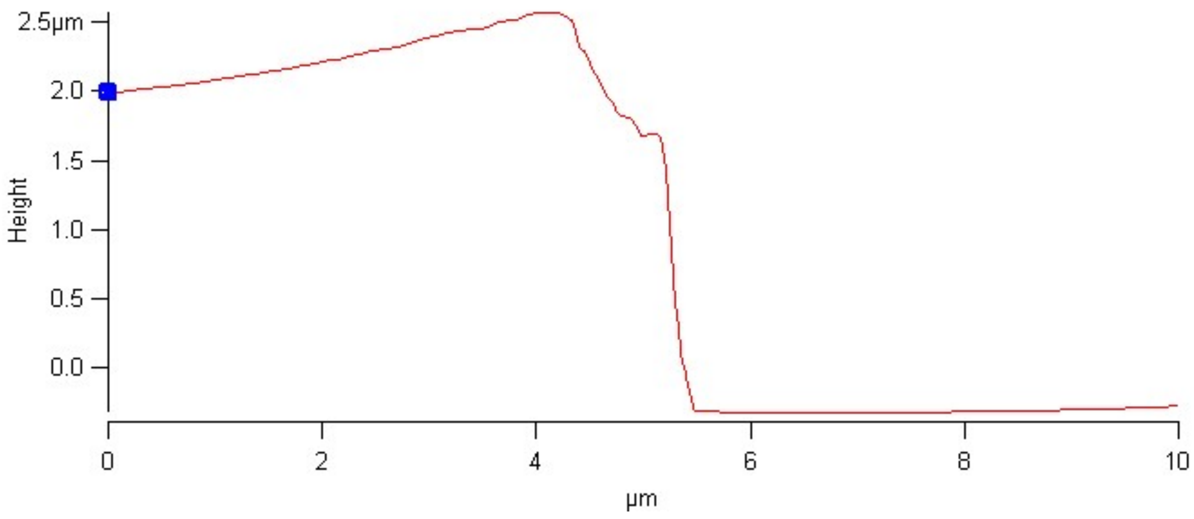

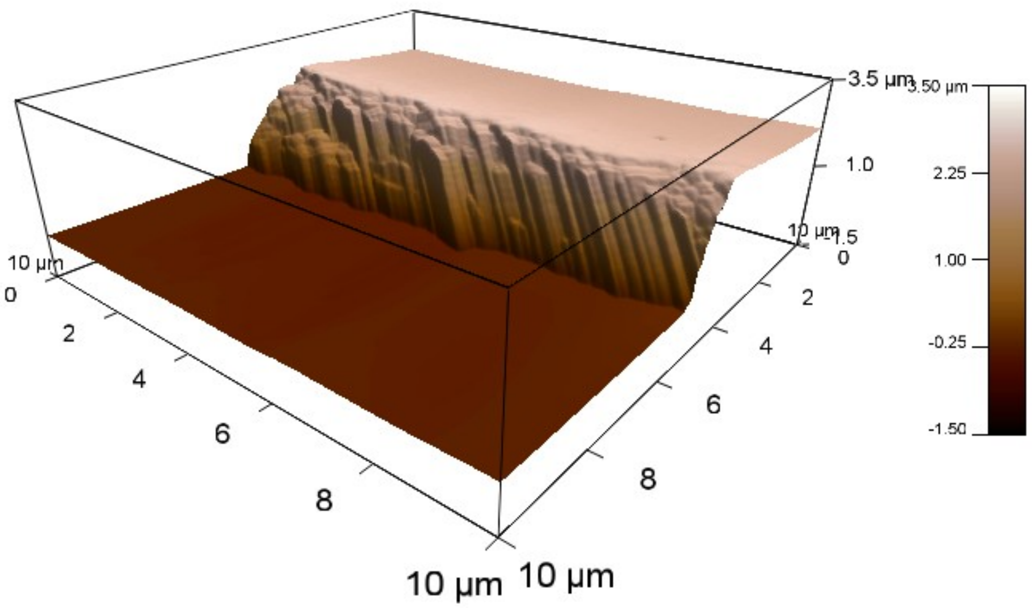

Figure S16: AFM measurements of a cut PAMB layer on a HMDS-coated glass substrate, prepared under the same conditions as the PE-supported PAM films, but without corona pre-treatment.

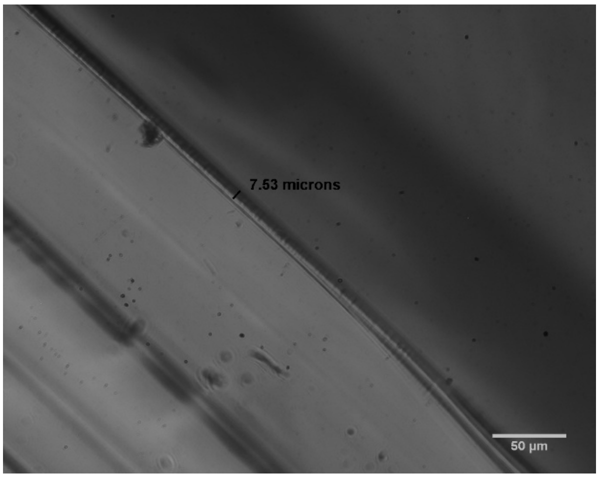

(a) side view of glass slide with PAMB layer

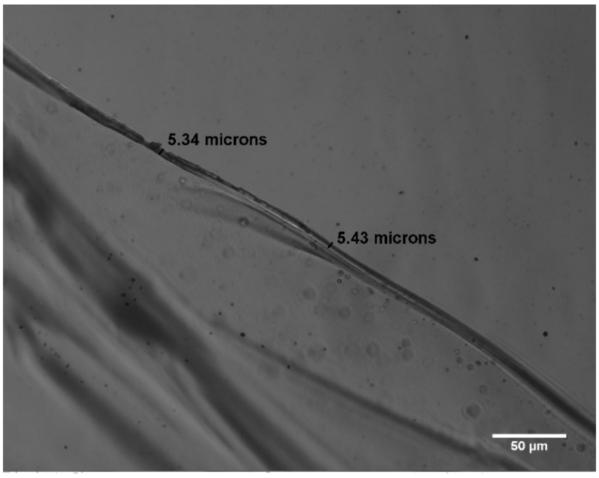

(b) side view of PAMB layer only

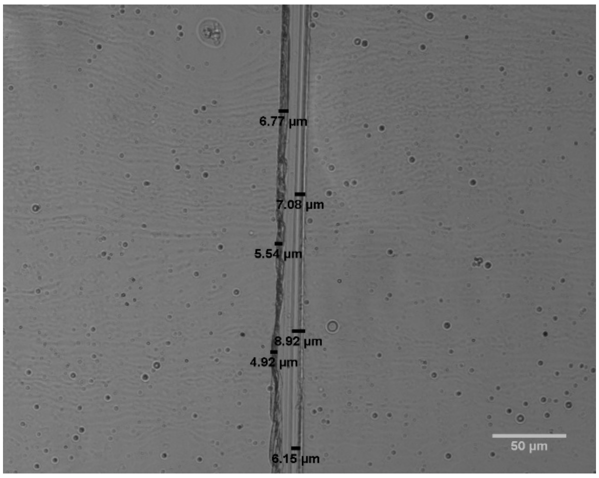

(c) top view of cut PAMB layer

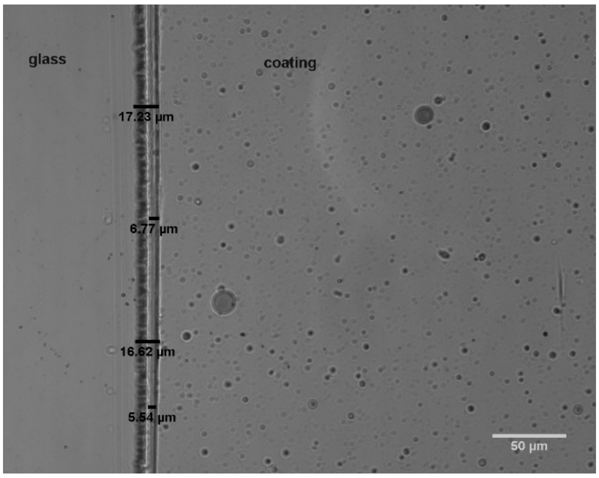

(d) top view of PAMB layer edge

Figure S17: Optical micrographs of PAMB layers on HMDS-coated glass substrates, prepared under the same conditions as the PE-supported PAM films, but without corona pre-treatment.
